# Screening, Safety Assessment, and Process Optimization of Lactic Acid Bacteria from Traditional Yak Yogurt as Adjunct Cultures

**DOI:** 10.3390/microorganisms14030630

**Published:** 2026-03-11

**Authors:** Weiming Shuang, Xiaodong Zeng, Ting Li, Jing Li, Qing Sun, Lianhong Chen

**Affiliations:** 1 College of Pharmacy and Food, Southwest Minzu University, Chengdu 610225, China; wms0901@outlook.com (W.S.); 250951352013@stu.swun.edu.cn (X.Z.); lt15685707637@outlook.com (T.L.); lijingaccepted@126.com (J.L.); 2 School of Foreign Languages and Literatures, Southwest Minzu University, Chengdu 610041, China; sunnysunq@163.com

**Keywords:** *Limosilactobacillus fermentum*, yak yoghurt, adjunct starter, cheese ripening, whole-genome sequencing, safety assessment, Gouda cheese, process optimization

## Abstract

Cheese ripening is slow and costly, driving interest in accelerating maturation. This study aimed to isolate a safe, efficient adjunct starter from traditional Sichuan yak yoghurt, a niche rich in stress-adapted lactic acid bacteria. From 295 isolates, 15 strains tolerant to high salt, low pH, and low temperature were selected. Using acidification, autolysis, proteolysis, and peptidase activity as indices, principal component analysis identified *Limosilactobacillus fermentum* 270 as the best candidate. Phenotypic assays showed no haemolysis, gelatin liquefaction, indole production, or amino acid decarboxylase activity. Whole-genome sequencing confirmed species identity and revealed 52 protease/peptidase genes, complete pathways for diacetyl/acetoin biosynthesis and branched-chain amino acid conversion, and no functional biogenic amine synthesis genes. Stress-related genes (F-ATPase, glycine-betaine transport, cold-shock proteins) support cheese adaptability. Antibiotic resistance gene homologs were mainly chromosomal and unlinked to mobile genetic elements; a functional CRISPR-Cas system lowers horizontal transfer risk. The strain was developed as a freeze-dried direct-vat starter (97.3% viability). Orthogonal optimisation of yak Gouda cheese-making defined best conditions: 0.018% adjunct, 45 min acidification, pH 5.8, and 30% curd washing. *L. fermentum* 270 thus combines proteolytic, flavour-enhancing, genetic safety, and processing traits, offering a promising adjunct for accelerated cheese ripening.

## 1. Introduction

Cheese is a dairy product typically produced by the acidification and coagulation of milk, primarily through the action of fermenting agents [1]. More than 130 countries and regions worldwide produce cheese, with over 2000 identified varieties [2]. The distinctive flavor, texture and rich nutrient content of cheese—including proteins, lipids, vitamins and minerals—arise from prolonged ripening, a process that involves complex microbial activity, biochemical conversions and physical changes [3]. However, extended ripening increases production costs and the risk of microbial contamination, posing practical challenges for manufacture and commercialization [4]. Consequently, practical methods to accelerate ripening are of considerable interest.

Several strategies have been used to shorten maturation. Modifying ripening temperatures can accelerate biochemical development, as shown by Ceruti et al., who obtained Reggianito cheeses with 6-month-equivalent characteristics within 4 months by altering early-ripening conditions [5]. Addition of exogenous proteolytic materials (e.g., protease-rich plant pastes) has been reported to increase soluble nitrogen and free fatty acids and shorten ripening times [6]. High-pressure processing can also enhance proteolysis and reduce maturation time, although it may affect texture and flavor [7]. Supplementation with selected non-starter lactic acid bacteria can boost free amino acid accumulation during ripening and thus promote maturation [8]. Each approach can accelerate ripening but carries drawbacks: higher temperatures risk spoilage organisms, exogenous enzymes are difficult to control precisely, and physical treatments often incur high costs or alter product quality.

Using adjunct cultures is a comparatively safe, industry-compatible means to accelerate ripening: adjunct strains can be introduced without changing core manufacturing processes and primarily act by enhancing proteolytic and lipolytic pathways to enrich flavor complexity and nutritional quality [9]. An ideal adjunct must remain metabolically active under typical ripening stresses (low temperature, high salt, and acidity) and, via a robust protease system and autolysis, effectively drive proteolysis to accelerate flavor formation [10]. Extreme or unique ecological niches are promising sources of microbes with exceptional stress tolerance and metabolic traits. Yak milk is characterized by high nutritional value, containing 4.0–6.5% protein, 5.6–7.5% fat, and abundant minerals such as calcium and iron, along with a rich profile of unsaturated fatty acids and bioactive peptides [11]. Beyond its nutritional composition, metagenomic studies have revealed that yak milk harbors a unique microbial ecosystem, with fermentation processes significantly enriching beneficial lactic acid bacteria such as *Lactobacillus delbrueckii* and *Streptococcus thermophilus*, which are closely associated with flavor formation and metabolic functions [12]. In this context, traditional yak yogurt from the western Sichuan Plateau presents a valuable reservoir: its traditionally handmade natural fermentation by local herders, combined with the high-altitude, cold, and high-UV environment, likely selects for strains with enhanced environmental resilience, while the fermentation process itself enriches microbes adapted to dairy substrates and capable of protein and lipid metabolism [13].

Here, we report a targeted screening of 295 lactic acid bacteria isolates from traditional western Sichuan yak yogurt under simulated cheese ripening conditions (high salt, low temperature, acidic pH). Candidates were re-screened for protease production, acidification, autolysis, proteolytic degradation and total peptidase activity; high-scoring strains were subjected to comprehensive safety tests and genomic analysis. Our aim is to identify a safe, effective adjunct culture and to optimize its application parameters for industrial cheese production.

## 2. Materials and Methods

### 2.1. Preliminary Screening of Adjunct Cultures for Cheese

All 295 lactic acid bacteria (LAB) strains used in this study were previously isolated from traditional yak yogurt in western Sichuan and stored in our laboratory. Frozen strains (−80 °C) were activated by twice subculturing in de Man, Rogosa and Sharpe (MRS) medium (Hope Bio-Technology Co., Ltd., Qingdao, China) with a 2% (*v*/*v*) inoculum to ensure viability. Following a modified version of the method described by Benavente Beltrán et al., 1 mL of activated culture was inoculated into MRS medium adjusted to simulate cheese-ripening conditions (MRS supplemented with 4% NaCl (Tianjin Aopu Chemical Co., Ltd., Tianjin, China), pH adjusted to 5.0), incubated statically at 14 °C for 72 h, and optical density at 600 nm (OD_600_) was recorded [14].

### 2.2. Evaluation of Candidate Adjunct Culture

#### 2.2.1. Screening for Protease-Producing LAB

Activated cultures were streaked on MRS agar (Hope Bio-Technology Co., Ltd., Qingdao, China) to obtain single colonies. Individual colonies were picked with sterile toothpicks and spot-inoculated onto skim milk agar; plates were incubated at 37 °C for 24 h and inspected for clear proteolytic zones. The presence of a clear halo around colonies was taken as evidence of extracellular protease production.

Preparation of skim milk agar: prepare a 5% (*w*/*v*) skim milk medium (Anchor, Fonterra Co-operative Group Ltd., Auckland, New Zealand; sterilized at 115 °C for 20 min) and MRS agar (Hope Bio-Technology Co., Ltd., Qingdao, China; sterilized at 121 °C for 20 min) separately; when both media cool to ~50 °C, mix at a 1:1 (*v*/*v*) ratio to obtain a final medium containing 2.5% skim milk.

#### 2.2.2. Determination of Acidification Capacity

Acidification ability was determined with minor modifications to Cavanagh et al. Activated cultures were inoculated at 1% (*v*/*v*) into sterile 10% skim milk (prepared with skim milk powder from Anchor, Fonterra Co-operative Group Ltd., Auckland, New Zealand) and incubated at 30 °C for 24 h. The change in pH (ΔpH) between inoculation and 24 h was used to represent acidification capacity [15].

#### 2.2.3. Determination of Autolysis Rate

Autolysis was determined according to the method described by Fernandes et al., with minor modifications [16]. Activated cells were harvested by centrifugation at 10,000× *g* for 10 min at 4 °C, washed twice, and resuspended in 0.02 M phosphate-buffered saline (PBS, Beijing Solarbio Science & Technology Co., Ltd., Beijing, China; pH 6.8). Cell suspensions were adjusted to an absorbance at 650 nm (A_650_) of 0.60–0.80, which was recorded as A_0_. The suspensions were then incubated statically at 37 °C for 24 h, after which the absorbance at 650 nm was measured again (A_24_). PBS was used as a blank control. Autolysis (%) was calculated according to the following equation:

(1) $$Autolysis(\%)=(1-A_{24}/A_{0})\times 100$$

#### 2.2.4. Proteolytic Activity (Measurement of Amino Nitrogen)

Proteolytic activity was evaluated by determining amino nitrogen content according to the Chinese national standard GB 5009.235-2016 [17]. Activated cultures were inoculated at 2% (*v*/*v*) into 12% skim milk and incubated at 37 °C for 24 h. A 5.0 g aliquot of fermented sample was mixed with 60 mL distilled water and titrated with 0.1 M NaOH (Tianjin Aopu Chemical Co., Ltd., Tianjin, China) to pH 8.2 (volume V_1_). Subsequently, 20 mL of neutral formaldehyde solution (Chengdu Jinshan Chemical Reagent Co., Ltd., Chengdu, Sichuan, China) was added, and titration was continued to pH 9.2 (volume V_2_). A blank titration volume (V_0_) was recorded under the same conditions. The amino nitrogen content (x, expressed as nitrogen mass fraction) was calculated using the following formula:

(2) $$x=\frac{0.1\times (V_{2}-V_{1}-V_{0})\times 0.014}{5.000}\times 100$$

#### 2.2.5. Determination of Total Peptidase Activity

Total peptidase activity was determined using a modified cadmium–ninhydrin assay. A leucine standard curve (0.2–2.0 mM) was prepared. Total peptidase activity was determined using a modified cadmium–ninhydrin assay. A leucine standard curve (0.2–2.0 mM) was prepared. The substrate was 1% casein hydrolysate (Biosharp, Hefei, China) prepared by rennet (commercially available, analytical grade) hydrolysis at 30 °C for 24 h in 0.05 M sodium citrate buffer (Tianjin Aopu Chemical Co., Ltd., Tianjin, China; pH 5.4) containing 0.2% sodium azide (analytical grade). Cell-free crude enzyme extract was prepared from cultures grown in MRS broth by PBS washing, ice-cold sonication and centrifugation. A 500 μL aliquot of crude enzyme was mixed with 500 μL substrate and incubated at 37 °C for 24 h. After reaction, 2 mL cadmium–ninhydrin reagent (ninhydrin purchased from Beijing Solarbio Science & Technology Co., Ltd., Beijing, China; other components were of analytical grade) was added and samples were heated at 84 °C for 5 min. Absorbance was measured at 507 nm. Enzyme activity was calculated from the leucine standard curve and expressed as leucine equivalents.

### 2.3. Principal Component Analysis (PCA) Methodology

To select strains suitable as cheese adjunct cultures, acidification capacity, autolysis, proteolytic ability and total peptidase activity were used as variables. PCA was performed using SPSS 27 to compute composite scores for the re-screened strains; strains with the highest composite scores were selected for subsequent analyses.

### 2.4. Safety Assessment Procedures

#### 2.4.1. Gelatin Liquefaction Assay

Gelatinase activity was assessed by stab inoculation of strains into gelatin medium. Cultures were incubated at 37 °C for 36 h and then chilled at 4 °C for 30 min. Liquefaction of the medium was recorded as a positive result. Staphylococcus aureus was used as a positive control.

#### 2.4.2. Indole Assay

Tryptophanase activity was detected using the Ehrlich–Böhme reagent. Strains were inoculated into peptone water (commercially available, analytical grade) and incubated at 37 °C for 48 h. Ether extraction was performed using diethyl ether (Chengdu Jinshan Chemical Reagent Co., Ltd., Chengdu, China) and indole reagent (commercially available, analytical grade) was added to the organic phase; development of a rose-red color in the ether layer was interpreted as positive.

#### 2.4.3. Hemolysis Assay

Activated cell suspensions were streaked onto Columbia blood agar (Hope Bio-Technology Co., Ltd., Qingdao, China) and incubated for 48 h. Hemolytic activity was assessed by observing clear zones around colonies. *Staphylococcus aureus* ATCC 6538 (Hope Bio-Technology Co., Ltd., Qingdao, China) served as a positive control.

#### 2.4.4. Determination of Amino Acid Decarboxylase Activity

Lysine, arginine, and ornithine decarboxylase activities were assessed using commercial decarboxylase test tubes (Hope Bio-Technology Co., Ltd., Qingdao, China). Single colonies were picked from MRS agar plates and inoculated into the corresponding decarboxylase media, overlaid with sterile mineral oil, and incubated at 37 °C for 24 h. A color change in the medium from yellow to purple was recorded as a positive result.

Histidine and tyrosine decarboxylase activities were determined according to the method described by He et al. with minor modifications [18]: MRS broth was used as the basal medium and supplemented with 0.01% (*w*/*v*) L-histidine hydrochloride (Shanghai Yuanye Bio-Technology Co., Ltd., Shanghai, China) or 0.01% (*w*/*v*) L-tyrosine disodium salt (Shanghai Yuanye Bio-Technology Co., Ltd., Shanghai, China), together with 0.006% (*w*/*v*) bromocresol purple (Shanghai Yuanye Bio-Technology Co., Ltd., Shanghai, China) as a pH indicator. The pH was adjusted to 5.0–5.3 to prepare the indicator media. Activated strains were inoculated at 1% (*v*/*v*) and incubated statically under anaerobic conditions at 37 °C for 72 h. A color change from yellow to purple was considered indicative of positive decarboxylase activity.

#### 2.4.5. Antibiotic Susceptibility Assay

Antibiotic susceptibility was determined by the disk diffusion (Kirby–Bauer) method. Activated strains were subcultured twice, adjusted to approximately 10^7^ colony-forming units per milliliter (CFU/mL), and 100 μL was spread evenly on MRS agar (Hope Bio-Technology Co., Ltd., Qingdao, China) plates. Plates were pre-equilibrated at 4 °C for 1 h, antibiotic disks (Jin’ao Technology & Chemical Co., Ltd., Qianjian, China) were applied, and plates were incubated inverted at 37 °C for 48 h. Inhibition zone diameters for seven antibiotics were measured with a caliper. Interpretation criteria are provided in Table S1.

### 2.5. Genomic Analysis Methodology

#### 2.5.1. Genome Sequencing, Assembly and Quality Control Procedures

Genomic DNA (gDNA) of the target strain was extracted using an E.Z.N.A.^®^ kit (Omega Bio-tek, Inc., Norcross, GA, USA). One microgram of gDNA was fragmented by sonication to 300–500 bp and libraries were prepared using the Illumina TruSeq^TM^ Nano DNA Library Prep Kit (Illumina, Inc., San Diego, CA, USA). Libraries were polymerase chain reaction (PCR)-enriched (8 cycles), gel-purified, quantified and sequenced on an Illumina NovaSeq 6000 platform (Illumina, Inc., San Diego, CA, USA) with PE150 chemistry. Raw reads were quality-filtered with Trimmomatic (v0.36). De novo assembly was performed with ABySS (v2.2.0). Assembly quality (completeness and contamination) was assessed using CheckM (v1.2.4) based on bacterial marker genes. The whole-genome sequencing data have been submitted to NCBI under BioProject PRJNA1335852, BioSample SAMN52025697 and assembly accession JBRIKF000000000.

#### 2.5.2. Protocol for Molecular Identification and Phylogenetic Analysis

16S rRNA gene-based phylogenetic analysis was used for molecular identification. Reference 16S sequences of related type strains were retrieved from NCBI. Multiple sequence alignment was performed using the MUSCLE algorithm integrated into MEGA 12; alignment ends were trimmed. Model testing indicated the Kimura 2-parameter model with Gamma distribution and Invariant Sites (K2 + G + I) as optimal. A maximum-likelihood tree was constructed in MEGA 12 and node support was evaluated by 1000 bootstrap replicates.

#### 2.5.3. Genome Annotation

Functional annotation was performed as follows: Gene Ontology (GO) and Clusters of Orthologous Groups (COG) annotations were assigned using eggNOG-mapper (v5.0) by comparing predicted proteins to the eggNOG database under default parameters. KEGG pathway annotation and KO assignment were performed using kofamscan (v1.2.0), which employs hidden Markov models (HMMs) for profile-based assignment, under default parameters.

#### 2.5.4. Screening of Target Functional Genes

Based on genome annotation, a keyword-based search was used to identify genes related to acid tolerance (e.g., ATP synthase, glutamate decarboxylase), salt tolerance (e.g., betaine transporters, potassium transporters), low-temperature tolerance (e.g., cold shock proteins, RNA helicases), and proteolysis (proteases/peptidases). Identified genes and their annotations were compiled for downstream interpretation.

#### 2.5.5. Annotation of Virulence Factor via VFDB

DIAMOND (v0.9.22.123) in blastp mode was used to search the Virulence Factor Database (VFDB) with an E-value cutoff of 1 × 10^−5^, minimum identity 70% (--id 70) and minimum subject coverage 90% (--subject-cover 90). Hits meeting these thresholds were considered high-confidence virulence factor matches.

#### 2.5.6. Bioinformatic Screening for Decarboxylase Genes

Representative, experimentally validated decarboxylase protein sequences were retrieved from NCBI (e.g., arginine decarboxylase XHA04458.1; lysine decarboxylase NP_418555.1; ornithine decarboxylase XHA00950.1; histidine decarboxylase WP_057828021.1; tyrosine decarboxylase WP_002355450.1). These references cover major biogenic amine synthesis pathways. BLASTP (v2.12.0+) searches of the *L. fermentum* 270 predicted proteome were performed using two E-value thresholds (1 × 10^−^^5^ for high confidence and 0.1 for relaxed detection). No homologs meeting these criteria were detected.

#### 2.5.7. Annotation of Antibiotic Resistance Genes and Mobilome Analysis

ARG annotation was conducted using BLASTP (v2.9.0+) against the Comprehensive Antibiotic Resistance Database (CARD, v3.2.8) with an E-value cutoff of 1 × 10^−50^ and an initial identity filter of ≥50%. To evaluate horizontal transfer potential, MOB-suite (v3.1.9) was used to predict plasmid-associated scaffolds (MOB-cluster classification and Mash-based nearest-neighbor analysis) [19]. MobileElementFinder (v1.0.3) was used to detect other mobile genetic elements under two stringency settings (high: coverage ≥ 90%, identity ≥ 95%; low: coverage ≥ 80%, identity ≥ 85%) [20]. The genomic locations of ARGs were extracted from the genome annotation files for subsequent neighborhood analysis. For visualization of local genomic context, custom Python (v3.9) scripts extracted genes ±10 kb around target ARGs and Matplotlib (v3.10.7) was used to plot gene arrangements to assess proximity to mobile elements.

#### 2.5.8. Prophage Region Prediction

PhiSpy (v4.2.21) was used to predict prophage regions on assembled contigs with default parameters (minimum contig length 5000 bp; window size 30 genes; at least 5 genes required to call a prophage region) [21]. Prophage predictions were cross-referenced with CARD and VFDB annotations to evaluate whether ARGs or virulence factors reside within prophage regions and to assess potential roles in horizontal gene transfer.

#### 2.5.9. CRISPR Annotation

CRISPR arrays were identified using MinCED (v0.4.2) with parameters set to increase sensitivity for short arrays (search window length = 7; minimum repeat count = 2) [22].

### 2.6. Optimization of Yak-Milk Gouda Production

#### 2.6.1. Viable Count Assay

Lactic acid bacteria were enumerated following the national standard method for microbiological examination of food (GB 4789.35-2016) [23].

#### 2.6.2. Preparation of Yak-Milk Gouda Cheese

Yak milk was pasteurized at 63 °C for 30 min, cooled to 32 °C, and inoculated with 0.006% (*w*/*v*) commercial starter culture (TCC-3, containing *Lactobacillus delbrueckii* subsp. *bulgaricus* and *Streptococcus thermophilus*). After acidification, 0.01% (*w*/*v*) CaCl_2_ and 0.005% (*w*/*v*) calf rennet were added and coagulation proceeded at 32 °C for 30 min. Curd was cut into ~1 cm^3^ pieces, rested, then warmed to 37 °C with gentle stirring to expel whey; an equal volume of warm water was added and stirred for 30 min. The curd was drained, pressed and molded, then pressed at 50× mass for 10 h, flipped and pressed for an additional 8 h. Cheese blocks were brined at 14 °C in 20% (*w*/*v*) salt solution for 8 h and subsequently ripened at 14 °C and 90% relative humidity.

#### 2.6.3. Single-Factor Experiments

Single-factor experiments were conducted to evaluate the effect of individual process variables on cheese yield. The baseline conditions were set as follows: adjunct starter culture addition at 0.012% (*w*/*v*), acidification time at 60 min, pre-acidification pH at 5.6, and curd washing volume at 30% (*v*/*v*). While keeping three factors constant at their baseline levels, the fourth factor was varied across the following ranges: adjunct starter culture addition: 0.004, 0.006, 0.012, 0.018, 0.020% (*w*/*v*); acidification time: 30, 45, 60, 75, 90 min; pre-acidification pH: 5.2, 5.4, 5.6, 5.8, 6.0; curd washing volume: 10, 15, 30, 45, 50% (*v*/*v*). The optimal range identified for each factor from these experiments was used to define the levels for the subsequent orthogonal array design.

#### 2.6.4. Orthogonal Optimization of Yak-Milk Gouda Processing

Based on the outcomes of the single-factor experiments, an L_9_(3^4^) orthogonal array design was employed to optimize the key processing parameters for yak milk Gouda cheese. The four investigated factors were: adjunct starter culture addition (*w*/*v*), acidification time (min), pre-acidification pH, and curd washing volume (*v*/*v*). The specific factor levels and the complete experimental layout are provided in Tables S2 and S3, respectively. For all trials, a control group containing only the commercial starter culture was included. The experimental groups received the same commercial starter supplemented with the adjunct culture at the levels specified by the orthogonal design.

### 2.7. Statistical Analysis

All experiments were performed in triplicate. Data are presented as mean ± standard deviation. Statistical analyses were performed with SPSS 27; figures were produced with Origin 2023. Differences were considered significant at *p* < 0.05.

## 3. Results

### 3.1. Preliminary Screening of Adjunct Cultures

The OD_600_ values of the 295 lactic acid bacteria strains incubated under simulated cheese ripening conditions (high salt, low temperature, acidic pH) are listed in Table S4. Of the 295 strains, 46 strains (15.59%) exhibited OD_600_ < 0.20; the largest group (155 strains, 52.54%) had OD_600_ values in the 0.20–0.25 range; 72 strains (24.41%) showed OD_600_ between 0.25 and 0.30; 14 strains (4.75%) had OD_600_ in the 0.30–0.50 interval; and 8 strains (2.71%) reached OD_600_ > 0.50. Based on these results, the 15 strains that demonstrated the most stable growth under the simulated ripening conditions were selected for subsequent experiments (Table 1).

### 3.2. Re-Screening Results of Adjunct Cultures

#### 3.2.1. Protease Production

**Figure 1:**
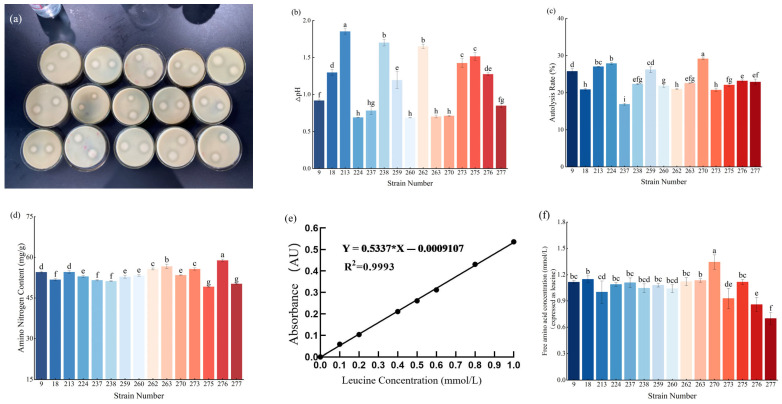
Secondary screening results of adjunct starter cultures. (**a**) Protease production capacity of the candidate strains. (**b**) Acid production capacity of the 15 strains. (**c**) Autolysis of the 15 strains. (**d**) Protein degradation capability of the 15 strains. (**e**) Standard curve of leucine. (**f**) Leucine standard curve and total peptidase activity of the 15 strains. Values with different lowercase letters (a–i) indicate significant differences among strains (*p* < 0.05).

As shown in Figure 1a, all 15 candidate LAB strains from the preliminary screen produced obvious colony halos and clear zones on skim-milk agar, indicating that each of the 15 strains secreted extracellular proteases and thus qualified for further re-screening.

#### 3.2.2. Acidification Capacity

Adjunct cultures for cheese should not have excessively high acidification activity, because over-acidification can negatively affect cheese flavor while accelerating ripening [24]. As shown in Figure 1b, among the 15 candidate strains, strain 213 exhibited very strong acidification (pH change, ΔpH = 1.85), which was significantly higher than that of the other strains. Seven strains showed ΔpH < 1. Strains 260, 9, 263, 270 and 237 had relatively weak acidification and therefore represent promising candidates for development as adjunct cultures.

#### 3.2.3. Autolysis Rate

Strains 9, 213, 224, 259 and 270 displayed relatively high autolysis, each exceeding 25% (Figure 1c). Strain 270 showed the highest autolysis (29.15%), a trait that is critical during cheese ripening because autolysis of LAB releases intracellular peptidases that further degrade peptides generated from protein hydrolysis into smaller peptides and free amino acids—key precursors of cheese flavor [25]. These results indicate that strain 270 may have substantial potential to promote flavor development.

#### 3.2.4. Proteolytic Activity (Amino Nitrogen)

Higher amino-nitrogen content indicates greater peptide bond cleavage and higher free amino acid levels. During cheese ripening, adjunct cultures can improve texture and flavor by protein degradation [26]. All 15 candidate strains exhibited measurable proteolytic activity (Figure 1d). The top five strains by proteolytic capacity were: strain 276 (58.90 ± 0.45 mg/g), strain 263 (56.69 ± 0.81 mg/g), strain 262 (55.73 ± 0.34 mg/g), strain 273 (55.70 ± 0.43 mg/g) and strain 9 (54.57 ± 0.35 mg/g). The weakest proteolytic activity was observed for strain 275 (49.17 ± 0.18 mg/g).

#### 3.2.5. Total Peptidase Activity

A leucine standard curve was constructed with leucine concentration on the *x*-axis and absorbance on the *y*-axis (Figure 1e). The regression equation was y = 0.5337x − 0.0009107 (R^2^ = 0.9993), showing excellent linearity. All 15 cell-free extracts exhibited measurable peptidase activity, and strain 270 showed significantly higher peptidase activity than the other strains (Figure 1f).

### 3.3. Principal Component Analysis (PCA)

Principal component analysis (PCA) was performed using four variables for the 15 candidate strains: acidification capacity, autolysis rate, proteolytic ability and total peptidase activity. As shown in Table S5, three components had eigenvalues greater than 1, with variance contribution rates of 36.226%, 27.498% and 25.124%, respectively. The cumulative contribution of these three principal components reached 88.848%, indicating that they collectively capture the major variation among the screened strains; therefore, these three components were retained for further analysis.

The component score expressions (Table S6) were as follows: Y_1_= −0.527 X_1_ + 0.571 X_2_ − 0.110 X_3_ + 0.273 X_4_; Y_2_ = 0.330 X_1_ + 0.310 X_2_ + 0.782 X_3_ + 0.305 X_4_; Y_3_= 0.358 X_1_ − 0.142 X_2_ − 0.415 X_3_ + 0.821 X_4_. Where X_1_, X_2_, X_3_, and X4 represent acidification capacity, autolysis rate, proteolytic ability (amino nitrogen), and total peptidase activity, respectively. A composite score Y was calculated as the weighted sum of the three principal component scores using their relative contributions: Y = 36.226%/88.848% Y_1_ + 27.498%/88.848%Y_2_ + 25.124%/88.848% Y_3_. After standardizing the raw data and applying the composite-score formula, the comprehensive scores for all strains were obtained (Table S7). Strain 270 achieved the highest composite score, followed by strains 263 and 9, indicating their strong potential as adjunct cultures to promote cheese ripening. Notably, strain 270 exhibited the highest autolysis rate and total peptidase activity among the 15 strains, while strains 263 and 9 showed relatively high proteolytic ability and peptidase activity. Based on these results, the three top-scoring strains were selected for subsequent safety evaluation.

### 3.4. Safety Assessment

#### 3.4.1. Gelatin Liquefaction Test

**Figure 2:**
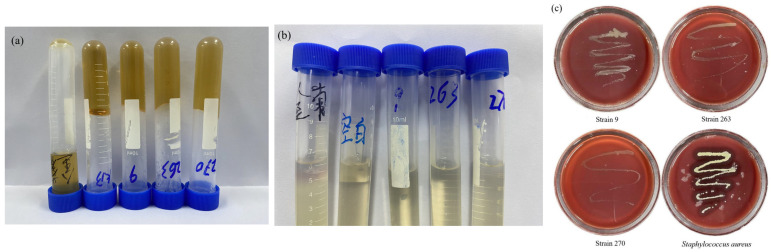
Gelatin liquefaction, indole, and hemolysis tests. (**a**) Gelatin liquefaction test; (**b**) Indole test; (**c**) Hemolysis test. In panels (**a**,**b**), the samples are arranged from left to right as follows: *Staphylococcus aureus* (positive control), negative control, Strain 9, Strain 263, and Strain 270.

Gelatinase is a zinc metalloprotease encoded by the gelE gene and may act as a virulence factor by promoting tissue invasion [27]. As shown in Figure 2a, none of the candidate strains (9, 263 and 270) caused gelatin liquefaction; their results matched the negative control. By contrast, the positive control (*Staphylococcus aureus*) produced clear liquefaction of the gelatin medium, indicating gelatinase activity.

#### 3.4.2. Indole Test

Indole production reflects tryptophanase activity and may be associated with metabolic interference or toxicity when present in excess. As shown in Figure 2b, the ether layer of the positive control developed a pink color, while the negative control and strains 9, 263 and 270 were indole-negative, indicating that the tested LAB strains do not produce detectable indole under the assay conditions.

#### 3.4.3. Hemolysis Test

Hemolysis is an important phenotypic indicator of bacterial virulence. Staphylococcus aureus is a typical β-hemolytic organism [28]. As shown in Figure 2c, no hemolytic zones were observed around colonies of strains 9, 263 or 270, whereas S. aureus exhibited clear β-hemolysis. These results indicate absence of hemolytic activity in the three candidate LAB strains.

#### 3.4.4. Amino Acid Decarboxylase Activity

**Figure 3:**
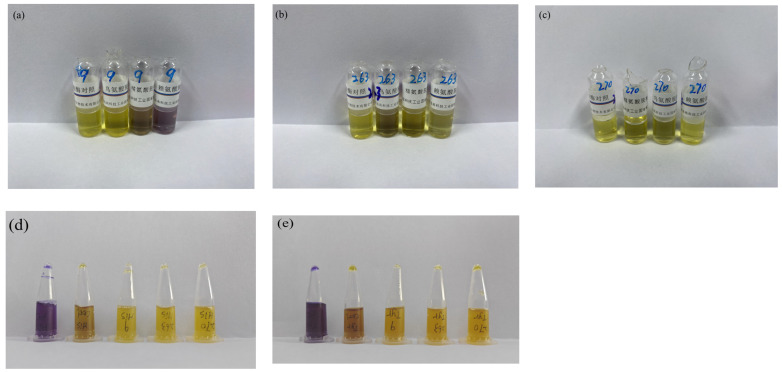
Amino acid decarboxylase test results. (**a**–**c**) Lysine, arginine, and ornithine decarboxylase tests for Strain 9 (**a**), Strain 263 (**b**), and Strain 270 (**c**), respectively. (**d**) Histidine decarboxylase test for the three strains. (**e**) Tyrosine decarboxylase test for the three strains.

Biogenic amines can cause multiple adverse effects in humans when ingested in excess, including migraine, headache, elevated blood pressure and fever; in severe cases they may lead to pulmonary edema and heart failure [29]. As shown in Figure 3, Strain 9 tested positive for lysine decarboxylase but was negative for ornithine, arginine, histidine and tyrosine decarboxylase activities. Lysine decarboxylation produces cadaverine, a compound with cytotoxic properties that can react with nitrite to form carcinogenic nitrosamines and may potentiate the toxicity of other biogenic amines by inhibiting their degradation. Under the colorimetric broth assay conditions used here, Strains 263 and 270 were negative for decarboxylase activity toward all tested amino acids. These results provide key preliminary safety evidence, indicating that no relevant biogenic-amine–producing enzyme activities were detected for Strains 263 and 270 under the current experimental conditions.

#### 3.4.5. Antibiotic Susceptibility Testing

Reports of antibiotic-resistant lactic acid bacteria have increased in recent years; because resistance genes can be horizontally transferred to pathogens and pose public-health risks, antibiotic susceptibility screening and genomic analyses are essential when selecting and applying new LAB strains [30]. As shown in Table 2, all three strains were sensitive to penicillin, tetracycline, azithromycin and erythromycin, and resistant to kanamycin, vancomycin and norfloxacin. Previous studies indicate that many LAB display intrinsic resistance to certain aminoglycosides (e.g., gentamicin, kanamycin, neomycin, streptomycin), glycopeptides (e.g., vancomycin), fluoroquinolones (e.g., ciprofloxacin) and trimethoprim [31].

In summary, Strain 9’s lysine decarboxylase positivity places it at a disadvantage in terms of safety compared with Strains 263 and 270. Combined with the principal component analysis (in which Strain 270 achieved the highest composite score), Strain 270 was selected for subsequent studies.

### 3.5. Genomic Analysis of the Target Strain

#### 3.5.1. Genome Sequencing, Assembly and Quality Assessment

The genome of Strain 270 was sequenced on an Illumina NovaSeq 6000 platform (2 × 150 bp, paired-end). Raw reads were quality-filtered using Trimmomatic (v0.36). De novo assemblies were generated with ABySS (v2.2.0) using multiple k-mer values to optimize contiguity, and GapCloser (v1.12) was applied for scaffold gap filling and base correction. Assembly statistics are summarized in Table 3: the assembly comprised 117 scaffolds with a total length of 2,029,436 bp. Assembly contiguity was good, with scaffold N50 = 37,772 bp and N90 = 10,106 bp. The genomic GC content was 51.75%, consistent with values reported for members of the *Limosilactobacillus*/*Lactobacillaceae* group. The proportion of ambiguous bases (Ns) was extremely low (0.004%), indicating high base-calling accuracy.

CheckM quality assessment indicated high completeness (97.41%) and negligible contamination (0.00%), confirming that the assembly is of high quality and suitable for downstream functional gene analyses.

**Figure 4:**
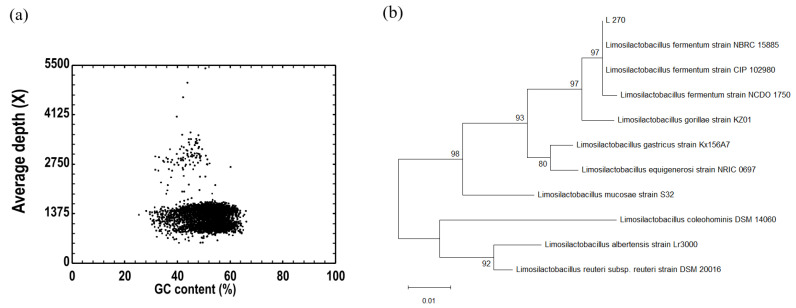
Genomic and phylogenetic analyses. (**a**) Correlation analysis between sample GC content and sequencing depth. (**b**) Phylogenetic tree based on the 16S rRNA gene sequence of Strain 270.

In addition, GC content versus sequencing depth analysis (Figure 4a) showed an approximately Poisson-like distribution of GC content across the assembly; points at high depth correspond to putative plasmid contigs, supporting the absence of significant contamination and the overall reliability of the assembly.

#### 3.5.2. Molecular Identification and Phylogenetic Analysis

Phylogenetic analysis based on 16S rRNA gene sequences (Figure 4b) placed Strain 270 within the *Limosilactobacillus fermentum* clade. The strain clustered closely with reference strains CIP 102980, NCDO 1750 and NBRC 15885, forming a well-supported clade. These molecular-level results corroborate the identification of Strain 270 as *Limosilactobacillus fermentum*.

#### 3.5.3. Protease and Peptidase Gene Annotation

Based on the whole-genome annotation of *Limosilactobacillus fermentum* 270 (hereinafter referred to as *L. fermentum* 270) (Table S8), 52 protease and peptidase genes were identified. These genes primarily represent protease/peptidase systems commonly found in lactic acid bacteria, including aminopeptidases (e.g., *pepN*, *pepC*), ATP-dependent proteases of the AAA+ family (e.g., *clpB*, *clpE*, *clpX*, *clpP*, *ftsH*), peptidoglycan-related proteases (e.g., *mrcA*, *dacA*), peptidases specific for small peptides (e.g., *pepX*, *pepT*, *pepQ*), and signal peptidases (e.g., *lepB*, *lspA*).

#### 3.5.4. Acid, Salt and Cold Tolerance Gene Annotation

Fifteen genes associated with stress tolerance were annotated in the *L. fermentum* 270 genome (Table S9). Acid-tolerance loci include eight F-type ATP synthase subunit genes (*atpA*, *atpD*, *atpB*, *atpG*, *atpH*, *atpF*, *atpC*, *atpE*). Three cold-tolerance-related genes were also identified, including a DEAD-box ATP-dependent RNA helicase (*cshB*) and cold-shock-like proteins (containing *cspA*). Additionally, four salt-tolerance genes were annotated, including those encoding glycine betaine/carnitine ABC transporter components (*opuC*, *opuBD*) and a potassium uptake protein (*trkA*/*ktrA*).

#### 3.5.5. KEGG-Based Analysis of Flavor-Formation Metabolic Potential

**Figure 5:**
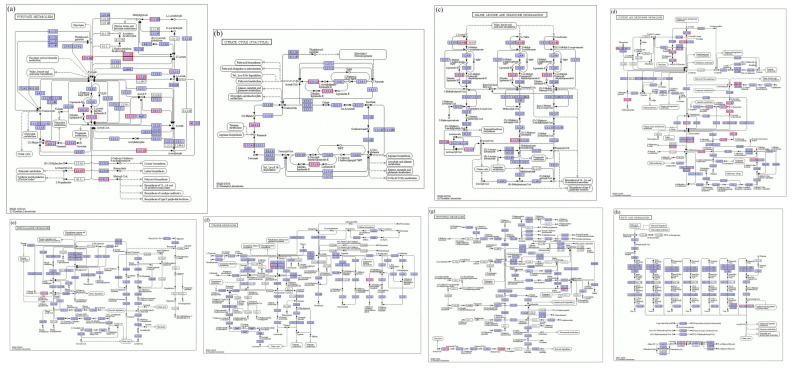
KEGG pathway analysis of flavor formation potential in *L. fermentum* 270. (**a**) Pyruvate metabolism (ko00620). (**b**) Citrate cycle (TCA cycle) (ko00020). (**c**) Lysine degradation (ko00310). (**d**) Cysteine and methionine metabolism (ko00270). (**e**) Phenylalanine metabolism (ko00360). (**f**) Tyrosine metabolism (ko00350). (**g**) Tryptophan metabolism (ko00380). (**h**) Fatty acid degradation (ko00071). Genes highlighted in red indicate those functionally annotated in the genome of *L. fermentum* 270.

KEGG annotation was used to systematically evaluate core metabolic pathways in the *L. fermentum* 270 genome that are relevant to cheese flavor formation (Figure 5). In carbohydrate metabolism, the genome encodes complete gene sets for pyruvate metabolism (ko00620) and the citrate cycle (TCA, ko00020). Homologs to α-acetolactate decarboxylase (EC 4.1.1.5) and 2,3-butanediol dehydrogenase/acetoin dehydrogenase (EC 1.1.1.4/303) were identified. Regarding amino acid catabolism, the genome harbors a complete complement of genes for branched-chain amino acid degradation, including branched-chain amino acid transaminase (EC 2.6.1.42), dihydrolipoyl dehydrogenase (EC 1.8.1.4), and downstream β-oxidation-related enzymes. For sulfur-containing amino acids, homologs of cystathionine β-lyase (EC 4.4.1.13) were detected, while methionine γ-lyase (EC 4.4.1.11) was absent. Notably, no genes encoding tyrosine decarboxylase (EC 4.1.1.25) or tryptophan decarboxylase (EC 4.1.1.28) were detected in the aromatic amino acid metabolism pathways. With respect to lipid metabolism, fatty-acid degradation (ko00071) appears to rely primarily on generalist enzymes (e.g., alcohol dehydrogenases, acyl-CoA transferases) rather than high-activity, dedicated lipases.

#### 3.5.6. Virulence Factor Annotation (VFDB)

The genome of *L. fermentum* 270 was screened against the Virulence Factors Database (VFDB) [32]. Four homologs with similarity to known virulence-associated genes were returned, specifically *lisR*, *tuf*, *hasC*, and *eno* (summarized in Table 4). Importantly, canonical virulence determinants such as hemolysins, gelatinases (*gelE*), or tryptophanases were not detected in the genome.

#### 3.5.7. Bioinformatic Analysis of Decarboxylase Genes

Phenotypic decarboxylase tests for *L. fermentum* 270 were negative across the panel of amino acids. To corroborate this at the genetic level, representative, experimentally validated decarboxylase protein sequences were used as queries in BLASTP (v2.12.0+) searches of the predicted proteome. Searches were run under both stringent (E-value < 1 × 10^−5^) and relaxed (E-value < 0.1) thresholds; no significant homologs were detected under either criterion. The concordance of negative phenotype and absence of decarboxylase homologs provides strong evidence that *L. fermentum* 270 lacks the genetic capacity to synthesize putrescine, cadaverine, spermidine, histamine or tyramine—further supporting its food-safety profile for dairy applications.

#### 3.5.8. Antibiotic Resistance Gene (ARG) Annotation and Mobilome Analysis

**Figure 6:**
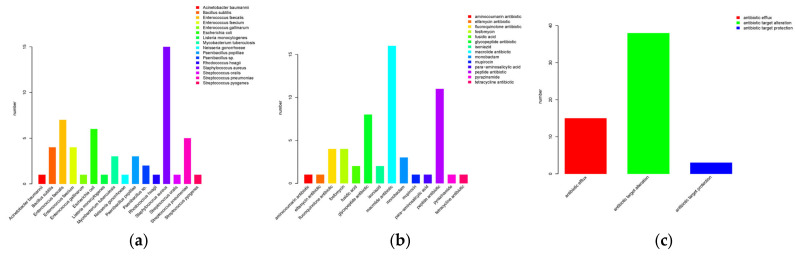
Summary of antibiotic resistance gene annotation. (**a**) Species-of-origin statistics. (**b**) Drug-class statistics. (**c**) Resistance-mechanism statistics.

BLASTP searches against the Comprehensive Antibiotic Resistance Database (CARD) identified 59 putative antibiotic-resistant homologs in the *L. fermentum* 270 genome. The species origin, drug-class distribution, and putative resistance mechanisms of these homologs are summarized in Figure 6. After applying an identity filter, 12 genes showed >50% sequence identity to known resistance determinants (Table 5), and among these, 6 exhibited >60% identity. Putative homologs with <50% identity are listed in Table S10. Canonical acquired, mobile ARGs such as tet, erm, or cat were not detected.

Genomic localization analysis of these 12 candidate genes revealed that three (L_270000057, L_270000058, L_270000041) reside on a single predicted plasmid scaffold (L_270_scaffold1). The remaining nine genes are chromosomally encoded (Table 6). MGE analysis identified multiple insertion sequences and composite transposons (Table S11). However, none of the candidate antibiotic resistance genes located on the chromosome were found to co-localize with any predicted MGEs.

**Figure 7:**
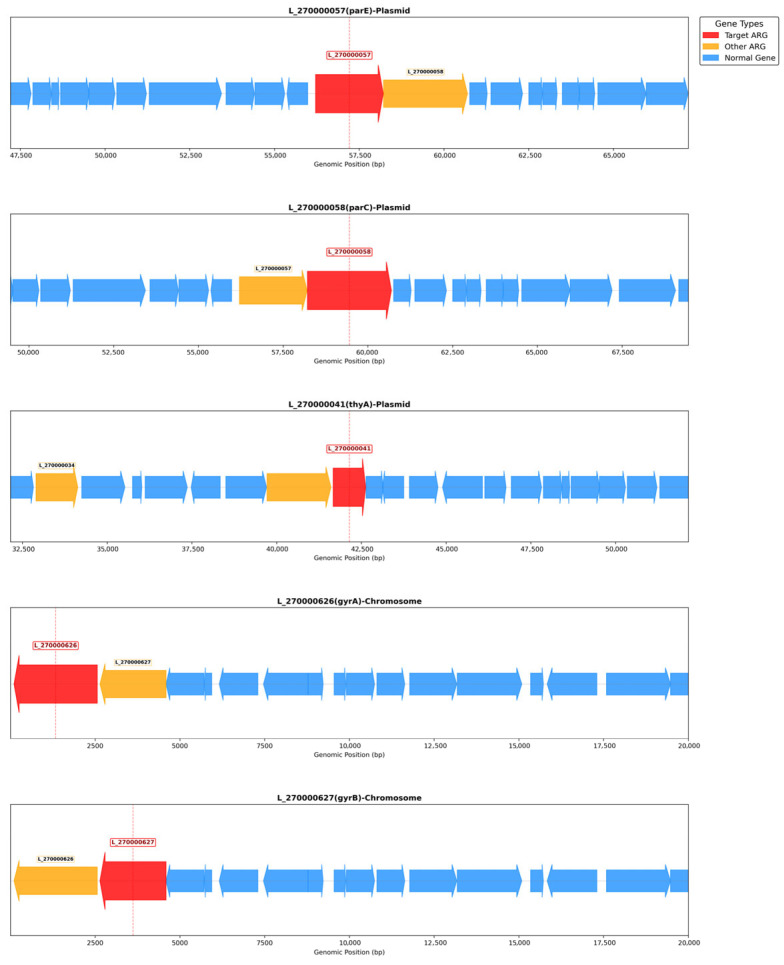
Genomic context of key antibiotic resistance genes. The diagram illustrates a 20-kb window (10 kb upstream and downstream) around each target gene. Genes are shown as arrows (orientation = transcriptional strand; length ∝ gene size). Color code: target ARG (red), other ARGs (orange), non-ARG genes (blue).

To further probe horizontal-transfer risk, we performed local genomic-context analyses for five key genes—the three plasmid-located candidate ARGs (L_270000057, L_270000058, L_270000041) and two chromosomal homologs associated with fluoroquinolone resistance (L_270000626, L_270000627). For each target, we extracted ±10 kb of flanking sequence and visualized gene arrangements (Figure 7). Across the 20-kb windows examined, no MGEs such as transposases or integrases were detected adjacent to these five targets. MobileElementFinder genome-wide scans corroborated this finding, indicating that these ARGs are separated from predicted MGEs by at least 10 kb in the current assembly.

MGE analysis identified multiple insertion sequences and composite transposons (Table S11). However, none of the high-priority antibiotic resistance genes located on the chromosome were found to co-localize with any predicted MGEs. This indicates that these chromosomal resistance genes are currently embedded in a low-mobility genetic context.

#### 3.5.9. Prophage-Region Bioinformatic Analysis

PhiSpy prediction identified 19 prophage regions in the *L. fermentum* 270 genome. To evaluate the potential of prophage-mediated horizontal gene transfer (HGT), six representative prophage regions were selected for in-depth analysis: five large prophages (>13 kb) and one smaller prophage (Prophage11, ≈5.4 kb) that was annotated to contain a homolog of a putative virulence-associated gene (Table 7). Among the prophages, four contained gene homologs of potential concern (summarized in Table 8). Specifically, Prophage7 harbors a *fusE* homolog with 53.37% amino acid identity to known resistance determinants. Prophage2 and Prophage6 contain candidate genes related to membrane lipid metabolism (*pgsA*) and a protein implicated in cell-wall biosynthesis, respectively; however, the similarity of these matches is below 50%. Prophage11 carries an eno homolog encoding enolase.

#### 3.5.10. CRISPR Annotation Analysis

CRISPR arrays were identified genome-wide using MinCED; five CRISPR loci were detected on five different scaffolds (Table 9). The CRISPR3 array, located on L_270_scaffold37, is the largest and most complete: it contains 30 highly conserved repeats (mean length ≈ 28 bp) and 29 spacers. The remaining four arrays are smaller, consisting of 2–4 repeats.

### 3.6. Preparation of Yak-Milk Gouda and Process Optimization

#### 3.6.1. Preparation and Viability of Lyophilized Adjunct Culture

To ensure the manufactured adjunct formulation was suitable for production, viable counts were measured before and after freeze-drying. Viable cell counts of the commercial starter TCC-3 were 11.12 ± 0.05 log CFU·g^−1^, and the *L. fermentum* 270 culture suspension was 10.98 ± 0.02 log CFU·g^−1^. After freeze-drying, the direct-to-vat adjunct preparation of *L. fermentum* 270 contained 10.68 ± 0.02 log CFU·g^−1^, corresponding to a survival rate of 97.27%. These results indicate that the strain retained high viability following lyophilization, supporting its suitability for industrial application.

#### 3.6.2. Single-Factor Experiment Results

**Figure 8:**
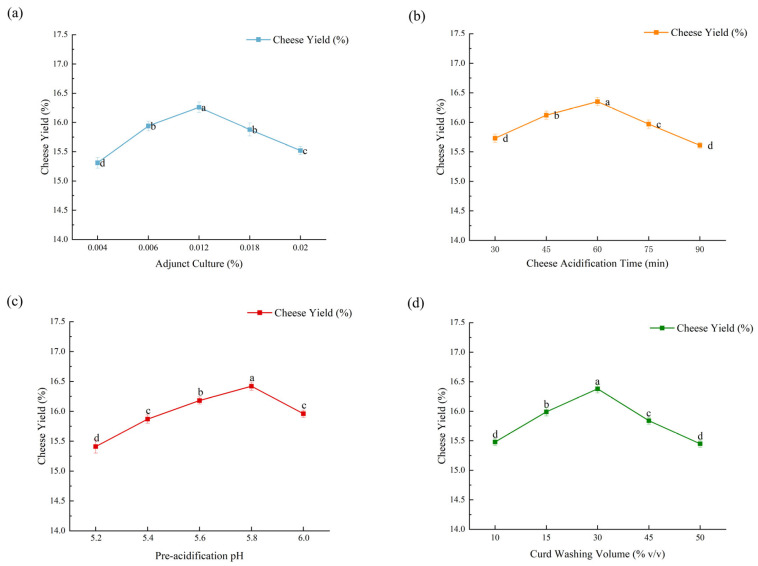
Effects of processing parameters on cheese yield: (**a**) adjunct starter culture addition; (**b**) cheese acidification time; (**c**) pre-acidification pH; and (**d**) curd washing volume. Different lowercase letters (a–d) above data points indicate significant differences among tested levels within each factor (*p* < 0.05).

Single-factor tests (Figure 8) produced unimodal responses for all variables. Cheese yield rose with increasing adjunct starter culture addition, reaching a maximum of 16.26% at 0.012% (*v*/*v*) and declining thereafter. A similar pattern was observed for acidification time, with peak yield (16.35%) at 60 min. Increasing pre-acidification pH produced an initial increase in yield followed by a decrease, with the highest yield (16.42%) at pH 5.8. Curd washing volume likewise exhibited a peak yield (16.38%) at 30% wash volume. Based on these trends, three levels per factor were selected for orthogonal optimization: adjunct starter culture addition = 0.006, 0.012, 0.018% (*v*/*v*); acidification time = 45, 60, 75 min; pre-acidification pH = 5.4, 5.6, 5.8; and curd washing volume = 15, 30, 45% (*v*/*v*).

#### 3.6.3. Orthogonal Experiment Results and Verification

Orthogonal L_9_(3^4^) experiments and ANOVA (Table 10 and Table 11) ranked the factors affecting yak-milk Gouda yield (descending): pre-acidification pH > adjunct starter culture addition > curd washing volume > acidification time. All four factors had highly significant effects on yield (*p* < 0.01). Range analysis produced a theoretical optimal combination of A_2_B_3_C_2_D_1_, but the highest observed experimental yield corresponded to combination A_3_B_1_C_3_D_2_.

A verification experiment (Table 12) showed that the experimental combination (A_3_B_1_C_3_D_2_) produced a higher cheese yield than the theoretical combination (A_2_B_3_C_2_D_1_). Therefore, the final optimized parameters for yak-milk Gouda were established as follows: adjunct starter culture addition = 0.018% (*w*/*v*), acidification time = 45 min, pre-acidification pH = 5.8, and curd washing volume = 30% (*v*/*v*).

## 4. Discussion

### 4.1. Environmental Adaptation of LAB Strains from the Yak Yogurt Niche

The selection of robust adjunct cultures is a critical step in ensuring their survival and metabolic activity during the protracted and often harsh cheese-ripening process. In this study, traditional yak yogurt from the western Sichuan Plateau served as a unique ecological niche for isolating high-performing lactic acid bacteria (LAB) strains. This extreme high-altitude environment, characterized by fluctuating temperatures and low pH, inherently selects for strains with superior environmental resilience compared to those from more temperate sources.

Our preliminary screening (Section 3.1) underscores the challenge of simulated cheese-ripening conditions (high salt, low temperature, and acidic pH). While the majority of the 295 tested strains (52.54%) exhibited only moderate growth (OD_600_ between 0.20 and 0.25), a very small fraction (only 2.71%) was able to reach OD_600_ values above 0.50. This wide distribution of growth phenotypes suggests that while many LAB can persist in dairy environments, only a select few possess the specialized physiological machinery required to thrive under the specific stressors of cheese maturation.

The exceptional performance of the top 15 selected strains, particularly *L. fermentum* 270, is deeply rooted in their genomic architecture. As revealed in Section 3.5.4, the presence of a comprehensive suite of 15 stress-tolerance genes provides the necessary molecular toolkit for this resilience. The eight F-type ATP synthase subunit genes (*atpA–H*) are essential for maintaining intracellular pH homeostasis through proton translocation, a mechanism previously reported to be vital for LAB survival in acidic dairy matrices [33]. Furthermore, the identification of cold-shock proteins (*cspA*) and RNA helicases (*cshB*) ex-plains the strain’s capacity to maintain translation and cellular metabolism at the low temperatures (14 °C) typical of ripening cellars [34]. Crucially, the presence of specific transporters for compatible solutes (*opuC*, *opuBD*) and potassium ions (*trkA*/*ktrA*) elucidates the strain’s ability to counteract osmotic pressure from high salt concentrations (4% NaCl). Collectively, the integration of phenotypic distribution data and genomic evidence strongly highlights that *L. fermentum* 270 is a highly adapted candidate, capable of maintaining the metabolic vigor necessary to function as an effective adjunct culture.

### 4.2. Genomic Integrity and Multi-Dimensional Safety Assessment

Ensuring the absolute safety of adjunct cultures is a fundamental prerequisite for their application in the food industry. Phenotypically, *L. fermentum* 270 exhibited an excellent safety profile, demonstrating no hemolytic activity, no gelatin liquefaction, and no indole production while also remaining sensitive to common clinical antibiotics (Section 3.4). This favorable phenotypic profile is robustly corroborated by our deep genomic assessment. Screening against the VFDB confirmed the complete absence of canonical acquired virulence determinants, such as hemolysins or gelatinases (*gelE*). Although four homologs with similarity to virulence-associated genes (*lisR*, *tuf*, *hasC*, and *eno*) were detected, contextual analysis reveals that these encode essential housekeeping enzymes or stress-response regulators (e.g., elongation factor Tu and enolase) rather than true toxins. Thus, the genotypic profile perfectly aligns with the negative phenotypic safety assays, supporting the non-pathogenic nature of this strain.

Beyond intrinsic toxicity, the absence of transferable antibiotic resistance is a critical safety criterion to prevent horizontal gene transfer (HGT) within the human microbiome. Although 59 putative ARG homologs were identified in the genome, the vast majority correspond to intrinsic, species-typical targets (e.g., *gyrA*, *parC*) rather than acquired resistance genes. Notably, canonical mobile ARGs (such as *tet*, *erm*, or *cat*) were not detected. Crucially, our genomic context analysis demonstrated that the identified candidate resistance genes are genomically isolated; they do not co-localize with any mobile genetic elements (MGEs), and no adjacent transposases or integrases were detected within a 20-kb window. Similarly, while 19 prophage regions were identified, the associated gene hits were predominantly low-identity homologs (e.g., *pgsA* [35] and cell-wall biosynthesis proteins [36] with <50% identity) or highly conserved core metabolic enzymes, indicating a negligible risk of prophage-mediated HGT. However, given the inherent limitations of short-read assemblies in precisely resolving plasmid structures and mobile elements [37], we adopt a conservative but scientifically rigorous interpretation: while current multi-level evidence strongly points to low mobility risk, definitive confirmation of plasmid dynamics will necessitate long-read sequencing in future studies.

Furthermore, the genomic stability and biosafety of *L. fermentum* 270 are significantly reinforced by its active defense mechanisms. The identification of a highly complete CRISPR array (CRISPR3) with substantial spacer diversity strongly indicates that the strain maintains an active CRISPR-Cas adaptive immune system. From a food safety perspective, this natural barrier provides the strain with an enhanced capacity to recognize and degrade invading plasmid or phage DNA [38]. This favorable genomic feature actively reduces the likelihood that the strain will acquire novel antibiotic-resistant genes or virulence factors from complex dairy matrices or gut environments. Overall, the integration of phenotypic susceptibility and comprehensive genomic surveillance firmly establishes the QPS (Qualified Presumption of Safety) potential of *L. fermentum* 270.

### 4.3. Proteolytic System and Metabolic Basis for Flavor Development

Beyond safety and survival, the development of distinct flavor profiles in cheese is deeply dependent on the metabolic network of adjunct cultures. Genomic analysis of *L. fermentum* 270 revealed an extensive repertoire of 52 protease and peptidase genes, providing a solid genetic basis for the proteolytic phenotype observed during preliminary screening. The abundant presence of ATP-dependent proteases (AAA+ family) indicates strong intracellular mechanisms for protein quality control and degradation [39], which aids the strain’s adaptation to the harsh cheese matrix. Specifically, the identification of aminopeptidase genes (such as *pepN* and *pepX*) is of great technological interest. As previously reported, these enzymes are essential for hydrolyzing bitter peptides and significantly contribute to the development of desirable cheese flavors [40]. Furthermore, the functional potential of these genes is well-supported by previous enzymatic research; for instance, Cristofolini et al. demonstrated that purified PepX retains functional stability under typical cheese-ripening conditions (e.g., at 5 °C and pH 4.5) [41].

In parallel, our KEGG-based metabolic analysis indicates that *L. fermentum* 270 encodes a coherent and complementary network that further drives flavor formation. In carbohydrate metabolism, the identification of α-acetolactate decarboxylase and 2,3-butanediol dehydrogenase constitutes the crucial biochemical route from pyruvate to diacetyl and acetoin—volatile compounds that contribute desirable buttery and creamy notes [42]. This aligns with the findings of Decadt et al., who demonstrated that adding specific LAB adjuncts in Gouda cheese significantly increased the levels of these compounds, reinforcing their role in enhancing dairy-fat-related flavor notes [43].

Additionally, the complete complement of genes for branched-chain amino acid degradation implies the capacity to generate short-chain fatty acids (e.g., isobutyric and isovaleric acids) associated with nutty and dried-fruit notes in mature cheeses [44]. Interestingly, the specific detection of cystathionine β-lyase and the absence of methionine γ-lyase suggest that sulfur-volatile production may proceed primarily via Strecker degradation. This metabolic routing potentially favors the formation of milder, more complex sulfur volatiles rather than single, intense off-flavors [45]. Regarding lipid metabolism, although the strain relies on generalist enzymes rather than dedicated lipases, it remains highly competent at transforming existing lipid-derived intermediates into secondary flavor compounds [46]. Crucially, the genomic absence of tyrosine and tryptophan decarboxylases perfectly corroborates the phenotypic safety assays, confirming the strain’s inability to synthesize biogenic amines of food-safety concern, thereby reinforcing its suitability as a flavor-augmenting adjunct.

### 4.4. Technological Feasibility and Industrial Potential for Yak-Milk Gouda

Translating the excellent metabolic potential and robust safety profile of *L. fermentum* 270 into practical industrial applications requires precise process control and high formulation stability. Notably, our lyophilization assay (Section 3.6.1) demonstrated that the direct-to-vat adjunct preparation of *L. fermentum* 270 maintained an outstanding survival rate of 97.27% after freeze-drying. This exceptional viability indicates remarkable resistance to the osmotic and cold stresses of industrial manufacturing, strongly supporting its suitability for commercial distribution.

Furthermore, the physicochemical properties of yak milk—specifically its characteristically high protein and fat content—create a highly structured curd matrix that demands specific fermentation parameters compared to standard bovine milk. Through our orthogonal optimization (Section 3.6.2 and Section 3.6.3), the ideal preparation conditions for yak-milk Gouda incorporating this adjunct culture were successfully established. Our results indicated that an optimal adjunct starter culture addition of 0.018% (*w*/*v*), an acidification time of 45 min, a pre-acidification pH of 5.8, and a curd washing volume of 30% (*v*/*v*) were crucial for achieving the maximum cheese yield (17.04%). Optimizing these specific parameters is of paramount importance, as they directly dictate the moisture retention, mineral solubilization, and ultimate textural integrity of the cheese matrix. Importantly, these optimized conditions ensure that *L. fermentum* 270 reaches its optimal metabolic activity, allowing its diverse array of peptidases and flavor-forming pathways (discussed in Section 4.3) to function efficiently during the ripening phase. Furthermore, although *L. fermentum* is inherently heterofermentative, its high autolytic capacity and the rapid depletion of fermentable sugars by the primary starter effectively limit excessive CO_2_ production during this late ripening stage, thereby preventing structural defects in the Gouda cheese matrix. Consequently, the application of this tailored process not only guarantees the standardization and maximum yield of yak-milk Gouda but also fully exploits the functional and sensory benefits conferred by this highly adapted adjunct culture. Ultimately, the establishment of these optimized process parameters lays a crucial foundation for future in-depth investigations into the specific biochemical ripening mechanisms driven by *L. fermentum* 270 in yak-milk cheese matrices.

## 5. Conclusions

This study successfully screened lactic acid bacteria isolated from traditional yak yogurt of western Sichuan under simulated cheese-ripening stress conditions (low temperature, high salt, low pH). Candidate strains were re-screened using key functional indicators—extracellular protease production, autolysis rate, proteolytic (amino-nitrogen) activity, and total peptidase activity—and ranked by principal component analysis. *Limosilactobacillus fermentum* 270 emerged as the most promising adjunct culture candidate. Comprehensive safety assessments strongly support its suitability for food applications: phenotypic tests indicated the absence of hemolysis, gelatinase activity, and indole production, with all assayed amino acid decarboxylase activities being negative. Whole-genome analyses corroborated these findings, revealing no functional amino acid decarboxylase genes or canonical virulence determinants. Although several antibiotic-resistant homologs were annotated, most are chromosomally located and not closely associated with predicted mobile genetic elements, suggesting a low horizontal-transfer risk; furthermore, the presence of an active CRISPR–Cas system supports robust genomic stability.

Functional genome annotation further underscores the strain’s application potential. *L. fermentum* 270 harbors a rich complement of protease and peptidase genes, and KEGG pathway analysis indicates complete routes for the synthesis of key dairy flavor compounds (e.g., diacetyl/acetoin) and branched-chain amino acid catabolism while lacking genes required for biogenic-amine biosynthesis. Collectively, these features indicate that *L. fermentum* 270 can accelerate ripening and enhance cheese flavor without compromising safety. Finally, we developed *L. fermentum* 270 into a direct-to-vat adjunct starter and applied it in yak-milk Gouda production. Orthogonal optimization identified practical processing parameters, and the lyophilized adjunct retained high viability (survival rate 97.27%), demonstrating excellent industrialization potential. In summary, *L. fermentum* 270 combines robust ripening-promoting properties, genetic safety, and process adaptability, making it a highly competitive candidate for development as a cheese adjunct culture. Future work using multi-omics approaches and controlled ripening trials is warranted to elucidate the strain’s mechanistic contributions during practical cheese maturation.

## Figures and Tables

**Table 1:** Dominant lactic acid bacteria in high salt, low temperature and acidic environment.

Strain ID	OD_600_
213	0.721 ± 0.037 ^a^
237	0.720 ± 0.010 ^a^
263	0.653 ± 0.015 ^b^
260	0.627 ± 0.018 ^bc^
262	0.601 ± 0.008 ^cd^
9	0.574 ± 0.011 ^d^
273	0.563 ± 0.033 ^d^
238	0.506 ± 0.020 ^e^
270	0.427 ± 0.011 ^f^
277	0.405 ± 0.011 ^fg^
259	0.397 ± 0.009 ^fgh^
224	0.367 ± 0.015 ^ghi^
276	0.356 ± 0.004 ^hi^
275	0.338 ± 0.044 ^ij^
18	0.311 ± 0.007 ^j^

Values with different lowercase letters (a–j) in the same column indicate significant differences (*p* < 0.05).

**Table 2:** Antibiotic susceptibility results.

Antibiotic	Strain 9		Strain 263		Strain 270	
	Inhibition Zone (mm)	Susceptibility	Inhibition Zone (mm)	Susceptibility	Inhibition Zone (mm)	Susceptibility
Penicillin (PEN)	31.85 ± 0.15	S	33.20 ± 0.20	S	31.80 ± 0.30	S
Kanamycin (KAN)	8.95 ± 0.35	R	10.97 ± 0.54	R	9.77 ± 0.26	R
Vancomycin (VAN)	8.15 ± 0.15	R	7.55 ± 0.15	R	8.20 ± 0.10	R
Tetracycline (TET)	20.20 ± 0.10	S	21.95 ± 0.05	S	20.15 ± 0.35	S
Azithromycin (AZM)	19.65 ± 0.15	S	20.33 ± 0.88	S	22.30 ± 0.60	S
Erythromycin (ERY)	29.40 ± 0.50	S	31.97 ± 0.61	S	27.85 ± 0.45	S
Norfloxacin (NOR)	6.85 ± 0.05	R	6.97 ± 0.12	R	8.90 ± 0.10	R

R, resistant; S, susceptible.

**Table 3:** Genome assembly quality assessment statistics of *L. fermentum* 270.

Assessment Item	Value
Number of scaffolds	117
Total length (bp)	2,029,436
Number of large scaffolds (>1 kbp)	95
Largest scaffold (bp)	120,508
Scaffold N50 (bp)	37,772
Scaffold N90 (bp)	10,106
GC content (%)	51.75
N rate (%)	0.004

**Table 4:** Summary of VFDB Annotation Results.

Gene ID	Identify (%)	Identify-Len	E-Value	Coverage (%)	Gene	VFid	Description	Type
L_270000308	71.9	228	9.2 × 10^−92^	100	lisR	VFG006826	Two-component response regulator	Regulation
L_270001292	70.6	391	3.0 × 10^−162^	98.73	tuf	VFG016490	Translation elongation factor Tu	Adherence/Invasion
L_270001585	70.9	302	2.4 × 10^−119^	99.66	hasC	VFG005874	UTP--glucose-1-phosphate uridylyltransferase	Immune Evasion
L_270002027	71.7	434	9.5 × 10^−178^	98.63	eno	VFG005582	Enolase	Enzyme

**Table 5:** Putative antibiotic resistance genes in *L. fermentum* 270 with >50% identity to entries in the CARD database.

Gene ID	Scaffold	Identify (%)	Identify-Len	E-Value	Coverage (%)	AROid	Gene	Drug Class	Resistance Mechanism
L_270001292	L_270_scaffold22	75.19	391	0	92.43	ARO:3003438	EF-Tu	elfamycin antibiotic	antibiotic target alteration
L_270000988	L_270_scaffold14	68.89	1186	0	100	ARO:3003285	rpoB	rifamycin antibiotic	antibiotic target alteration, antibiotic target replacement
L_270000996	L_270_scaffold14	68.5	692	0	99.86	ARO:3003735	fusA	fusidane antibiotic	antibiotic target alteration
L_270000989	L_270_scaffold14	66.64	1181	0	97.85	ARO:3003291	rpoC	peptide antibiotic	antibiotic target alteration
L_270000057	L_270_scaffold1	65.36	638	0	95.94	ARO:3003315	parE	fluoroquinolone antibiotic	antibiotic target alteration
L_270000627	L_270_scaffold7	62.68	635	0	98.76	ARO:3003301	gyrB	aminocoumarin antibiotic	antibiotic target alteration
L_270000626	L_270_scaffold7	57.78	829	0	93.46	ARO:3003296	gyrA	fluoroquinolone antibiotic	antibiotic target alteration
L_270000058	L_270_scaffold1	55.82	808	0	97.82	ARO:3003311	parC	fluoroquinolone antibiotic	antibiotic target alteration
L_270000308	L_270_scaffold3	55.51	227	3.00 × 10^−83^	100	ARO:3000838	arlR	disinfecting agents and antiseptics, fluoroquinolone antibiotic	antibiotic efflux
L_270001012	L_270_scaffold14	53.37	178	1.00 × 10^−61^	100	ARO:3003737	fusE	fusidane antibiotic	antibiotic target alteration
L_270001260	L_270_scaffold21	52.76	923	0	100	ARO:3003729	ileS	mupirocin-like antibiotic	antibiotic target alteration
L_270000041	L_270_scaffold1	52.09	311	2.00 × 10^−104^	100	ARO:3004153	thyA	salicylic acid antibiotic	antibiotic target alteration

**Table 6:** Genomic localization of candidate antibiotic-resistant genes.

Gene ID	Scaffold	Location Type
L_270001292	L_270_scaffold22	Chromosome
L_270000988	L_270_scaffold14	Chromosome
L_270000996	L_270_scaffold14	Chromosome
L_270000989	L_270_scaffold14	Chromosome
L_270000057	L_270_scaffold1	Plasmid
L_270000627	L_270_scaffold7	Chromosome
L_270000626	L_270_scaffold7	Chromosome
L_270000058	L_270_scaffold1	Plasmid
L_270000308	L_270_scaffold3	Chromosome
L_270001012	L_270_scaffold14	Chromosome
L_270001260	L_270_scaffold21	Chromosome
L_270000041	L_270_scaffold1	Plasmid

**Table 7:** Characteristics and genomic locations of six representative prophage regions in *L. fermentum* 270.

Prophage ID	Scaffold	Length (bp)	Gene Count	Contains Risk Genes
Prophage3	L_270_scaffold4	25,291	37	no
Prophage2	L_270_scaffold4	20,813	19	yes
Prophage7	L_270_scaffold14	17,373	31	yes
Prophage1	L_270_scaffold1	16,325	32	no
Prophage6	L_270_scaffold13	13,644	16	yes
Prophage11	L_270_scaffold61	5436	6	yes

**Table 8:** Putative virulence and antibiotic resistance gene homologs identified within prophage regions of *L. fermentum* 270.

Located Prophage	Gene ID	Gene Type	Gene	Identity (%)
Prophage7	L_270001012	Antibiotic Resistance Genes	fusE	53.37
Prophage2	L_270000414	Antibiotic Resistance Genes	pgsA	49.47
Prophage6	L_270000981	Antibiotic Resistance Genes	D-Ala-D-Ala	35.31
Prophage11	L_270002027	Virulence Factors	eno	71.70

**Table 9:** CRISPR annotation summary.

Seqid	CRISPR Id	Start	End	Repeat_Num	Aver_Repeat_Len	Spacer_Num	Aver_Spacer_Len
L_270_scaffold12	CRISPR1	1805	2049	4	36	3	33
L_270_scaffold30	CRISPR2	20,786	20,896	2	38	1	35
L_270_scaffold37	CRISPR3	18,889	20,685	30	28	29	33
L_270_scaffold51	CRISPR4	2561	2654	2	34	1	26
L_270_scaffold73	CRISPR5	3203	3304	2	26	1	50

**Table 10:** Analysis of orthogonal test results.

Group	Adjunct Starter Culture Addition/%	Acidification Time/min	Pre-Acidification pH	Curd Washing Volume/%	Cheese Yield/%
1	A_1_	B_1_	C_1_	D_1_	16.47 ± 0.11 ^bc^
2	A_1_	B_2_	C_2_	D_2_	16.28 ± 0.07 ^c^
3	A_1_	B_3_	C_3_	D_3_	16.29 ± 0.09 ^c^
4	A_2_	B_1_	C_2_	D_3_	16.32 ± 0.10 ^bc^
5	A_2_	B_2_	C_3_	D_1_	16.55 ± 0.05 ^b^
6	A_2_	B_3_	C_1_	D_2_	15.75 ± 0.11 ^e^
7	A_3_	B_1_	C_3_	D_2_	17.04 ± 0.13 ^a^
8	A_3_	B_2_	C_1_	D_3_	16.04 ± 0.16 ^b^
9	A_3_	B_3_	C_2_	D_1_	16.30 ± 0.09 ^c^
K_1_	43.10	83.4	85.1	88.4	
K_2_	44.06	86.8	87.2	87.4	
K_3_	41.03	87.0	86.4	85.6	
R	3.03	2.43	3.66	2.99	
k_1_	16.35	16.61	16.09	16.44	
k_2_	16.21	16.29	16.30	16.36	
k_3_	16.46	16.12	16.63	16.22	

Within a row, means with different superscripts differ significantly (*p* < 0.05).

**Table 11:** Variance analysis results of cheese yield rate in orthogonal test.

Factor	Sum of Squares	Mean Square	F-Value	*p*-Value
Adjunct starter culture addition	0.2929	0.14645	8.75	0.002
acidification time	1.1428	0.57140	34.14	0.000
Pre-acidification pH	1.3394	0.66969	40.01	0.000
Curd washing volume	0.2280	0.11401	6.81	0.006

**Table 12:** Verify experimental results.

Condition	Factor				Yield/%
	A	B	C	D	
Experimental set	A_3_	B_1_	C_3_	D_2_	17.04 ± 0.13
Theoretical set	A_2_	B_3_	C_2_	D_1_	16.61 ± 0.10

## Data Availability

The genome sequence data of *Limosilactobacillus fermentum* 270 have been deposited in the NCBI GenBank database under BioProject accession PRJNA1335852, BioSample accession SAMN52025697, and assembly accession JBRIKF000000000.

## Supplementary Materials

The following supporting information can be downloaded at: https://www.mdpi.com/article/10.3390/microorganisms14030630/s1, Table S1: Drug Sensitive Discs Drug Content and Resistance Judgment Criteria; Table S2: Orthogonal factor level table; Table S3: L9(3^4^) orthogonal test design table; Table S4: Growth of 295 lactic acid bacteria in high salt, low temperature and acidic environment; Table S5: Total variance interpretation; Table S6: Component score coefficient matrix; Table S7: Factor score table; Table S8: Annotation results of protease and peptidase genes; Table S9: Genes related to acid, salt, and low-temperature tolerance in *L. fermentum* 270; Table S10: Putative antibiotic resistance genes annotated in *L. fermentum* 270 against the CARD database; Table S11: Mobile Genetic Elements Identification Results.

## Abbreviations

The following abbreviations are used in this manuscript:

LAB	Lactic acid bacteria
MRS	de Man, Rogosa and Sharpe
OD	Optical density
PBS	Phosphate-buffered saline
PCA	Principal component analysis
WGS	Whole-genome sequencing
ARG	Antibiotic resistance gene
MGE	Mobile genetic element
VFDB	Virulence Factors Database
CARD	Comprehensive Antibiotic Resistance Database
CRISPR	Clustered regularly interspaced short palindromic repeats
HGT	Horizontal gene transfer
DVS	Direct vat set
