# Supplementary material for: Screening, Safety Assessment, and Process Optimization of Lactic Acid Bacteria from Traditional Yak Yogurt as Adjunct Cultures
Source: Microorganisms. 2026 Mar 11;14(3):630. doi: 10.3390/microorganisms14030630 (PMC13029014; doi:10.3390/microorganisms14030630)
Supplement: Supplementary file 1 [file microorganisms-14-00630-s001.zip › microorganisms-4181895-supplementary.pdf]

Table S1. Drug Sensitive Discs Drug Content and Resistance Judgment Criteria

Drug Name	Potency	Interpretation Criteria (Inhibition Zone Diameter: mm)		
		Susceptible (S)	Intermediate (I)	Resistant (R)
Penicillin (PEN)	10 U/disc	≥29	-	≤28
Kanamycin (KAN)	30 μg/disc	≥18	14-17	≤13
Vancomycin (VAN)	30 μg/disc	≥17	15-16	≤14
Tetracycline (TET)	30 μg/disc	≥15	12-14	≤11
Azithromycin (AZM)	15 μg/disc	≥18	14-17	≤13
Erythromycin (ERY)	15 μg/disc	≥23	14-22	≤13
Norfloxacin (NOR)	30 μg/disc	≥17	13-16	≤12

Note: R (Resistant), I (Intermediate), S (Susceptible).

Table S2. Orthogonal factor level table

Level	factor			
	Adjunct starter culture addition /%	acidification time /min	Pre-acidification pH	Curd washing volume /%
1	0.006	45	5.4	15
2	0.012	60	5.6	30
3	0.018	75	5.8	45

Table S3. L9(3^4) orthogonal test design table

Trial No.	A	B	C	D
	Adjunct starter culture addition /%	acidification time /min	Pre-acidification pH	Curd washing volume /%
1	0.006	45	5.4	15
2	0.006	60	5.6	30
3	0.006	75	5.8	45
4	0.012	45	5.6	45
5	0.012	60	5.8	15
6	0.012	75	5.4	30
7	0.018	45	5.8	30
8	0.018	60	5.4	45
9	0.018	75	5.6	15

Table S4. Growth of 295 lactic acid bacteria in high salt, low temperature and acidic environment

Strain ID	OD_{600}	Strain ID	OD_{600}	Strain ID	OD_{600}
1	0.353±0.021	24	0.235±0.024	48	0.227±0.021
2	0.269±0.016	25	0.200±0.016	49	0.210±0.024
3	0.290±0.008	26	0.216±0.030	50	0.224±0.014
4	0.268±0.012	27	0.227±0.016	51	0.208±0.008
5	0.296±0.009	28	0.237±0.005	52	0.248±0.020
6	0.260±0.017	29	0.212±0.015	53	0.204±0.013
7	0.303±0.031	30	0.225±0.029	54	0.231±0.018
8	0.276±0.016	31	0.234±0.007	55	0.264±0.024
9	0.574±0.011	32	0.204±0.017	56	0.169±0.005
10	0.275±0.021	33	0.219±0.024	57	0.248±0.028
11	0.243±0.019	34	0.195±0.012	58	0.216±0.011
12	0.247±0.030	35	0.221±0.011	59	0.204±0.014
13	0.280±0.019	36	0.201±0.024	60	0.250±0.023
14	0.271±0.016	37	0.202±0.021	61	0.241±0.031
15	0.253±0.021	38	0.206±0.018	62	0.229±0.021
16	0.303±0.016	39	0.208±0.023	63	0.184±0.009
17	0.249±0.021	40	0.213±0.022	64	0.210±0.012
18	0.311±0.007	41	0.234±0.029	65	0.194±0.018
19	0.243±0.028	43	0.207±0.006	66	0.227±0.003
20	0.234±0.022	44	0.211±0.015	67	0.204±0.031
21	0.217±0.016	45	0.232±0.021	68	0.178±0.005
22	0.271±0.020	46	0.247±0.009	69	0.182±0.011
23	0.212±0.008	47	0.214±0.015	70	0.259±0.014

Table S4 (continued).

Strain ID	OD_{600}	Strain ID	OD_{600}	Strain ID	OD_{600}
71	0.211±0.007	95	0.197±0.017	121	0.245±0.004
72	0.202±0.006	96	0.144±0.013	122	0.227±0.022
73	0.208±0.017	97	0.178±0.006	123	0.244±0.025
74	0.242±0.003	98	0.208±0.011	124	0.255±0.007
75	0.175±0.010	99	0.179±0.015	125	0.214±0.012
76	0.222±0.017	100	0.198±0.003	126	0.207±0.005
77	0.177±0.008	101	0.257±0.018	127	0.216±0.015
78	0.183±0.012	102	0.273±0.020	128	0.248±0.018
79	0.17±0.016	103	0.229±0.009	129	0.227±0.011
77	0.177±0.008	104	0.248±0.004	130	0.226±0.009
78	0.183±0.012	105	0.236±0.012	131	0.223±0.021
79	0.17±0.016	106	0.222±0.010	132	0.227±0.019
80	0.183±0.019	107	0.261±0.016	133	0.264±0.022
81	0.224±0.004	108	0.301±0.005	134	0.231±0.008
82	0.198±0.010	109	0.281±0.011	135	0.248±0.016
83	0.177±0.018	110	0.281±0.023	136	0.241±0.025
84	0.218±0.021	111	0.243±0.018	137	0.215±0.014
85	0.218±0.013	112	0.307±0.007	138	0.272±0.008
86	0.208±0.014	113	0.239±0.014	139	0.207±0.011
87	0.190±0.005	114	0.250±0.008	140	0.272±0.010
88	0.252±0.012	115	0.282±0.020	141	0.253±0.007
89	0.160±0.007	116	0.256±0.013	142	0.224±0.023
90	0.168±0.010	117	0.242±0.005	143	0.203±0.009
91	0.171±0.013	118	0.228±0.015	144	0.200±0.004
92	0.198±0.009	119	0.254±0.009	145	0.237±0.024
93	0.224±0.018	120	0.243±0.017	146	0.213±0.017
94	0.172±0.006	121	0.245±0.004	147	0.210±0.019

Table S4 (continued).

Strain ID	OD_{600}	Strain ID	OD_{600}	Strain ID	OD_{600}
148	0.203±0.004	175	0.198±0.013	203	0.256±0.017
149	0.221±0.015	176	0.203±0.009	204	0.290±0.026
150	0.199±0.008	177	0.197±0.002	205	0.266±0.014
151	0.276±0.015	178	0.257±0.021	206	0.227±0.008
152	0.285±0.018	179	0.193±0.016	207	0.251±0.017
153	0.265±0.023	180	0.171±0.008	208	0.233±0.013
154	0.263±0.005	181	0.250±0.014	209	0.258±0.021
155	0.308±0.017	182	0.240±0.022	210	0.278±0.026
156	0.258±0.007	183	0.277±0.019	211	0.233±0.009
157	0.196±0.004	184	0.182±0.005	212	0.251±0.015
158	0.238±0.013	185	0.230±0.012	213	0.721±0.037
159	0.265±0.022	186	0.251±0.017	214	0.255±0.019
160	0.291±0.016	187	0.254±0.010	215	0.226±0.005
161	0.215±0.012	188	0.271±0.021	216	0.220±0.013
162	0.256±0.018	189	0.254±0.013	217	0.207±0.004
163	0.227±0.008	190	0.216±0.004	218	0.199±0.011
164	0.265±0.016	191	0.216±0.019	219	0.185±0.009
165	0.246±0.007	193	0.189±0.007	220	0.183±0.007
166	0.261±0.017	194	0.180±0.003	221	0.212±0.012
167	0.269±0.022	195	0.256±0.016	222	0.197±0.008
168	0.276±0.014	196	0.225±0.011	223	0.221±0.002
169	0.241±0.008	197	0.178±0.008	224	0.367±0.015
170	0.193±0.003	198	0.192±0.012	225	0.211±0.016
171	0.182±0.011	199	0.241±0.020	226	0.220±0.014
172	0.196±0.010	200	0.216±0.013	227	0.214±0.008
173	0.191±0.004	201	0.282±0.024	228	0.212±0.011
174	0.212±0.007	202	0.234±0.018	229	0.269±0.019

Table S4 (continued).

Strain ID	OD_{600}	Strain ID	OD_{600}	Strain ID	OD_{600}
230	0.222±0.003	257	0.245±0.005	284	0.259±0.026
231	0.219±0.014	258	0.218±0.007	285	0.200±0.008
232	0.210±0.005	259	0.397±0.009	286	0.219±0.011
233	0.214±0.015	260	0.627±0.018	287	0.212±0.007
234	0.244±0.013	261	0.224±0.011	288	0.230±0.012
235	0.271±0.020	262	0.601±0.008	289	0.213±0.013
236	0.216±0.014	263	0.653±0.015	290	0.183±0.008
237	0.720±0.010	264	0.254±0.012	291	0.226±0.021
238	0.506±0.020	265	0.264±0.007	292	0.241±0.010
239	0.216±0.013	266	0.255±0.013	293	0.201±0.008
240	0.193±0.008	267	0.225±0.008	294	0.245±0.016
241	0.253±0.019	268	0.228±0.015	295	0.202±0.003
242	0.233±0.007	269	0.225±0.008	296	0.210±0.011
243	0.205±0.004	270	0.427±0.011	297	0.218±0.016
244	0.264±0.013	271	0.239±0.025		
245	0.150±0.005	272	0.248±0.024		
246	0.238±0.022	273	0.563±0.033		
247	0.261±0.018	274	0.256±0.017		
248	0.226±0.003	275	0.338±0.004		
249	0.250±0.016	276	0.356±0.005		
250	0.232±0.010	277	0.405±0.011		
251	0.274±0.011	278	0.203±0.004		
252	0.280±0.016	279	0.210±0.008		
253	0.248±0.011	280	0.217±0.010		
254	0.254±0.007	281	0.243±0.018		
255	0.209±0.009	282	0.239±0.015		
256	0.300±0.014	283	0.224±0.021		

Table S5. Total variance interpretation

Component	Initial eigenvalues			Initial eigenvalues		
	Total	% of Variance	Cumulative %	Total	% of Variance	Cumulative %
1	1.449	36.226	36.226	1.449	36.226	36.226
2	1.100	27.498	63.724	1.100	27.498	63.724
3	1.005	25.124	88.848	1.005	25.124	88.848
4	0.446	11.152	100.00			

Note: Extraction method: principal component analysis.

Table S6. Component score coefficient matrix

	Component 1	Component 2	Component 3
x_1	-0.527	0.330	0.358
x_2	0.571	0.310	-0.142
x_3	-0.110	0.782	-0.415
x_4	0.273	0.305	0.821

Table S7. Factor score table

Strain ID	F_1	F_2	F_3	Y	Rank
270	2.11607	0.77761	0.99878	3.89246	1
263	-1.02119	1.60575	0.57871	1.16327	2
9	0.78611	0.48866	-0.13533	1.13944	3
224	1.46089	-0.03268	-0.32281	1.105	4
259	0.52061	0.15559	0.16117	0.83737	5
262	-1.03026	0.97775	0.55414	0.50163	6
213	1.06808	0.20655	-1.02908	0.24555	7
18	-0.38216	-0.45261	1.03877	0.204	8
275	-0.38825	-1.02306	1.41765	0.00634	9
238	-0.79955	-0.36891	0.83351	-0.33495	10
260	0.31253	-0.58997	-0.39419	-0.67163	11
237	-0.48563	-1.39031	0.57844	-1.2975	12
276	-0.79028	1.30167	-1.85065	-1.33926	13
273	-1.14476	0.35942	-0.7124	-1.49774	14

Note :F_1=36.226%/88.848% Y_1； F_2=27.498%/88.848%Y_2 ； F_3=25.124%/88.848% Y_3

Table S8. Annotation results of protease and peptidase genes

Gene ID	Length (bp)	Assigned category	NR description (simplified)	SwissProt (simplified)	COG/KOG	Functional class	GO annotations (simplified, up to 6 items)	GO_count	GO_summary	KEGG_ID	KEGG_name
L_270000610	841	Peptidase	M1 family peptidase	Aminopeptidase N OS=... GN=pepN ...	COG0308	E	GO:0005575; GO:0005622; GO:0005623; GO:0005737; GO:0044424; GO:0044464	6	CC: cellular component; intracellular	-	-
L_270000986	834	Protease	ATP-dependent Clp protease ATP-binding subunit	Chaperone protein ClpB OS=... GN=clpB ...	COG0542	O	GO:0006950; GO:0008150; GO:0010035; GO:0010038; GO:0042221; ...	11	BP: stress response; biological processes	K03696	clpC
L_270001369	810	Peptidase	Xaa-Pro dipeptidyl-peptidase	Xaa-Pro dipeptidyl-peptidase OS=... GN=pepX ...	COG2936	E	-	0	-	K01281	pepX
L_270001208	754	Protease	Carboxypeptidase (fragment)	Penicillin-binding protein 1A (fragment) OS=... GN=ponA ...	COG0744	M	GO:0005575; GO:0005576	2	CC: cellular component	K05366	mrcA
L_270001648	748	Protease	ATP-dependent Clp protease ATP-binding subunit ClpE	ClpE OS=... GN=clpE ...	COG0542	O	GO:0005575; GO:0005623; GO:0005886; GO:0016020;	6	CC: cellular component; cell	K03697	clpE

L_270000559	698	Protease	ATP-dependent zinc metalloprotease FtsH	FtsH OS=... GN=ftsH ...	COG0465	O	GO:0044464; GO:0071944 GO:0003674; GO:0003824; GO:0004176; GO:0005575; GO:0005623; ...	34	MF/BP/CC: catalytic activity; proteolysis; intracellular	K03798	ftsH, hflB
L_270000294	694	Protease	Penicillin-binding protein transpeptidase	Penicillin-binding protein 2B OS=... GN=penA ...	COG0768	M	-	0	-	-	-
L_270000520	685	Protease	Clp protease ClpX (ATP-binding subunit)	Probable ATP-dependent Clp ATP-binding subunit OS=...	COG0542	O	-	0	-	K04086	clpL
L_270000671	634	Peptidase	M13 family peptidase	Neutral endopeptidase OS=... GN=pepO ...	COG3590	O	-	0	-	K07386	pepO
L_270001517	618	Protease	Peptidoglycan DD-metalloendopeptidase family protein	-	COG4942	D	-	0	-	-	-
L_270001877	602	Peptidase	Oligoendopeptidase F	Oligoendopeptidase F homolog OS=... GN=yjbG ...	COG1164	E	-	0	-	K08602	pepF, pepB
L_270001398	478	Peptidase	Dipeptidase	Probable dipeptidase OS=...	COG4690	E	-	0	-	K08659	pepDA, pepDB

L_270000361	472	Peptidase	Putative dipeptidase PepV	Putative dipeptidase SH1171 OS=... GN=SH1171 ...	COG0624	E	-	0	-	K01439	dapE
L_270000213	470	Peptidase	Dipeptidase	Probable dipeptidase OS=...	COG4690	E	-	0	-	K08659	pepDA, pepDB
L_270001121	456	Peptidase	Dipeptidase (probable A)	Probable dipeptidase A OS=... GN=pepDA ...	COG4690	E	-	0	-	K08659	pepDA, pepDB
L_270001344	445	Peptidase	Aminopeptidase C	Aminopeptidase C OS=... GN=pepC ...	COG3579	E	GO:0000096; GO:0000098; GO:0003674; GO:0003824; GO:0005575; ...	48	MF: catalytic activity; BP: proteolysis; CC: intracellular	K01372	BLMH, pepC
L_270000417	433	Protease	Zinc protease (uncharacterized)	Uncharacterized zinc protease YmfH OS=... GN=ymfH ...	COG0612	S	-	0	-	-	-
L_270001421	431	Protease	Peptidase S11 (D-Ala-D-Ala carboxypeptidase)	DacA OS=... GN=dacA ...	COG1686	M	GO:0003674; GO:0003824; GO:0004175; GO:0004180; GO:0004185; ...	29	MF: catalytic activity; BP: proteolysis; CC: cell / cell wall	K07258	dacC, dacA, dacD
L_270000418	424	Protease	Insulinase family protein (probable inactive metalloprotease)	Probable inactive metalloprotease YmfF OS=... GN=ymfF ...	COG0612	S	-	0	-	-	-
L_270000507	423	Protease	S1 family peptidase (HtrA-like)	Serine protease Do-like HtrA	COG0265	O	GO:0008150; GO:0009266;	4	BP: biological process; response to	-	-

				OS=... GN=htrA ...			GO:0009628; GO:0050896		temperature stimulus		
L_270000920	423	Protease	Zinc metalloprotease (eep-like)	Probable protease eep OS=... GN=eep ...	COG0750	M	GO:0000988; GO:0000989; GO:0003674; GO:0003824; GO:0004175; ...	71	MF/BP/CC: catalytic activity; proteolysis; cell/membrane -associated	K11749	rseP
L_270001541	417	Protease	D-Ala-D-Ala carboxypeptidas e (DacA)	DacA OS=... GN=dacA ...	COG1686	M	GO:0003674; GO:0003824; GO:0004175; GO:0004180; GO:0004185; ...	29	MF: catalytic activity; BP: proteolysis; CC: cell / cell wall	K07258	dacC, dacA, dacD
L_270001294	416	Protease	ATP-dependent Clp protease ATP-binding subunit ClpX	ClpX OS=... GN=clpX ...	COG1219	O	GO:0000166; GO:0000502; GO:0002020; GO:0003674; GO:0003824; ...	94	MF: nucleotide binding; BP: proteolysis; CC: intracellular	K03544	clpX, CLPX
L_270000019	404	Peptidase	Peptidase T	Peptidase T OS=... GN=pepT ...	COG2195	E	GO:0003674; GO:0003824; GO:0004177; GO:0005575; GO:0005622; ...	32	MF: catalytic activity; BP: proteolysis; CC: intracellular	K01258	pepT
L_270001626	375	Protease	CPBP family intramembrane metalloprotease	-	COG1266	S	-	0	-	-	-
L_270000277	373	Peptidase	Creatinase / Xaa-Pro dipeptidase (pepQ-like)	Xaa-Pro dipeptidase OS=... GN=pepQ ...	COG0006	E	-	0	-	-	-
L_270001770	372	Peptidase	Dipeptidase	Annotated as Aspartate aminotransfera	COG0436	E	-	0	-	-	-

				se (likely misannotation)								
L_270001571	367	Peptidase	Peptidase M24 family (pepQ)	Xaa-Pro dipeptidase OS=... GN=pepQ ...	COG0006	E	-	0	-	K01271	pepQ
L_270000781	342	Protease	Glutamate carboxypeptidase	-	COG0624 | COG3064	M	-	0	-	-	-
L_270001160	332	Protease	Protease (unknown)	-	COG0330	O	GO:0005575; GO:0005576; GO:0005618; GO:0005623; GO:0005886; ...	9	CC: cellular component; cell	-	-
L_270000952	326	Protease	Serine hydrolase (putative D-Ala-D-Ala carboxypeptidase-like)	Putative D-Ala-D-Ala carboxypeptidase OS=... GN=yfeW ...	COG1680	V	-	0	-	-	-
L_270002123	324	Protease	ISL3 family transposase / ATP-dependent protease annotation	ATP-dependent protease OS=...	COG3464	L	-	0	-	K07485	K07485
L_270001370	306	Peptidase	Proline iminopeptidase	Proline iminopeptidase OS=... GN=fpaP ...	COG0596	E	-	0	-	-	-
L_270001114	305	Protease	D-Ala-D-Ala carboxypeptidase / AmpH-like (PbpX-like)	Putative PbpX OS=... GN=pbpX ...	COG1680	V	-	0	-	-	-

L_270000573	299	Protease	Zinc metalloprotease HtpX	Protease HtpX homolog OS=... GN=htpX ...	COG0501	O	-	0	-	K03799	htpX
L_270000815	285	Peptidase	Type I methionyl aminopeptidase (MAP)	Methionine aminopeptidase 1 OS=... GN=map ...	COG0024	E	GO:0000096; GO:0003674; GO:0003824; GO:0004177; GO:0005488; ...	60	MF: catalytic activity; BP: protein processing & proteolysis; CC: intracellular	K01265	map
L_270000900	271	Peptidase	Peptidase (annotated)	Probable succinyl-DAP desuccinylase OS=... GN=dapE ...	COG0624	E	-	0	-	-	-
L_270002023	242	Protease	Clp protease ClpP (proteolytic subunit)	ATP-dependent Clp protease proteolytic subunit OS=... GN=clpP ...	COG0740	O (U)	-	0	-	K01358	clpP, CLPP
L_270000354	227	Protease	CPBP family intramembrane metalloprotease	-	COG1266	S	-	0	-	K07052	K07052
L_270000942	224	Peptidase	Peptidase M10	-	COG5549	O	-	0	-	-	-
L_270000292	221	Protease	Rhomboid family intramembrane serine protease	Rhomboid-like protease 3 OS=... GN=ROM3 ...	COG0705	S	-	0	-	K19225	gluP
L_270000643	203	Protease	Signal peptidase I (signal peptidase IB)	Signal peptidase IB OS=... GN=spsB ...	COG0681	U	-	0	-	K03100	lepB

L_270001377	199	Protease	L,D-transpeptidase (putative YciB)	Putative L,D-transpeptidase YciB OS=... GN=yciB ...	COG1376	M	-	0	-	-	-
L_270000835	197	Protease	CPBP family intramembrane metalloprotease	-	COG1266	S	-	0	-	K07052	K07052
L_270001465	196	Protease	Clp protease proteolytic subunit (ClpP)	ATP-dependent Clp protease proteolytic subunit OS=... GN=clpP ...	COG0740	O	GO:0003674; GO:0003824; GO:0004176; GO:0005488; GO:0005515; ...	73	MF: catalytic activity; BP: proteolysis; CC: intracellular	K01358	clpP, CLPP
L_270002117	165	Protease	Protease synthase / regulatory protein PAI1 (annotated)	Uncharacterized N-acetyltransferase in pepI region OS=...	COG0454 | COG0456	K	-	0	-	K22441	paiA
L_270000259	140	Protease	Signal peptidase (lspA)	Lipoprotein signal peptidase OS=... GN=lspA ...	COG0597	MU	-	0	-	K03101	lspA
L_270001566	119	Protease	Hypothetical / probable peptidoglycan endopeptidase LytE-like	Probable peptidoglycan endopeptidase LytE OS=... GN=lytE ...	COG0791 | COG1388	M	-	0	-	-	-
L_270001990	116	Protease	Hypothetical / Murein DD-endopeptidase MepH-like	Murein DD-endopeptidase MepH OS=... GN=mepH ...	COG0791 | COG5263	M	-	0	-	-	-
L_270000992	114	Peptidase	Prepilin peptidase	-	COG1989	N O U	-	0	-	-	-

L_270000993	63	Peptidase	Prepilin peptidase (short)	-	COG1989	N O U	-	0	-	-	-
L_270001474	50	Protease	Spore protease YyaC (from L. plantarum)	-	-	-	-	0	-	-	-
Gene ID	Length (bp)	Assigned category	NR description (simplified)	SwissProt (simplified)	COG/KOG	Functional class	GO annotations (simplified, up to 6 items)	GO_count	GO_summary	KEGG_ID	KEGG_name
L_270000610	841	Peptidase	M1 family peptidase	Aminopeptidase N OS=... GN=pepN ...	COG0308	E	GO:0005575; GO:0005622; GO:0005623; GO:0005737; GO:0044424; GO:0044464	6	CC: cellular component; intracellular	-	-
L_270000986	834	Protease	ATP-dependent Clp protease ATP-binding subunit	Chaperone protein ClpB OS=... GN=clpB ...	COG0542	O	GO:0006950; GO:0008150; GO:0010035; GO:0010038; GO:0042221; ...	11	BP: stress response; biological processes	K03696	clpC
L_270001369	810	Peptidase	Xaa-Pro dipeptidyl-peptidase	Xaa-Pro dipeptidyl-peptidase OS=... GN=pepX ...	COG2936	E	-	0	-	K01281	pepX
L_270001208	754	Protease	Carboxypeptidase (fragment)	Penicillin-binding protein 1A (fragment) OS=... GN=ponA ...	COG0744	M	GO:0005575; GO:0005576	2	CC: cellular component	K05366	mrcA

L_270001648	748	Protease	ATP-dependent Clp protease ATP-binding subunit ClpE	ClpE OS=... GN=clpE ...	COG0542	O	GO:0005575; GO:0005623; GO:0005886; GO:0016020; GO:0044464; GO:0071944	6	CC: cellular component; cell	K03697	clpE
L_270000559	698	Protease	ATP-dependent zinc metalloprotease FtsH	FtsH OS=... GN=ftsH ...	COG0465	O	GO:0003674; GO:0003824; GO:0004176; GO:0005575; GO:0005623; ...	34	MF/BP/CC: catalytic activity; proteolysis; intracellular	K03798	ftsH, hflB
L_270000294	694	Protease	Penicillin-binding protein transpeptidase	Penicillin-binding protein 2B OS=... GN=penA ...	COG0768	M	-	0	-	-	-
L_270000520	685	Protease	Clp protease ClpX (ATP-binding subunit)	Probable ATP-dependent Clp ATP-binding subunit OS=...	COG0542	O	-	0	-	K04086	clpL

Table S9. Genes related to acid, salt, and low-temperature tolerance in *L. fermentum* 270

Gene ID	Length(bp)	Function_Category	Key_Description	COG	COG_Function	GO_Classification	KEGG_Annotation
L_270000970	512	Acid Tolerance	ATP synthase subunit alpha	COG0056	C	MF: Molecular function, Catalytic activity; BP: Biological process, Metabolic process; CC: Cellular component, Intracellular	ATPF1A, atpA
L_270000972	473	Acid Tolerance	ATP synthase subunit beta	COG0055	C	-	ATPF1B, atpD
L_270000966	236	Acid Tolerance	ATP synthase subunit a	COG0356	C	MF: Molecular function, Catalytic activity; BP: Biological process, Metabolic process; CC: Cellular component, Intracellular	ATPF0A, atpB
L_270000971	287	Acid Tolerance	ATP synthase gamma chain	COG0224	C	CC: Cellular component, Cell	ATPF1G, atpG
L_270000969	181	Acid Tolerance	ATP synthase subunit delta	COG0712	C	BP: Biological process; CC: Cellular component, Cell	ATPF1D, atpH
L_270000968	168	Acid Tolerance	ATP synthase subunit b	COG0711	C	-	ATPF0B, atpF
L_270000973	140	Acid Tolerance	ATP synthase epsilon chain	COG0355	C	MF: Molecular function, Catalytic activity; BP: Biological process, Metabolic process; CC: Cellular component, Intracellular	ATPF1E, atpC
L_270000967	70	Acid Tolerance	ATP synthase subunit c	COG0636	C	MF: Molecular function, Catalytic activity; BP: Biological process, Metabolic process; CC: Cellular component, Intracellular	ATPF0C, atpE
L_270000112	292	Salt Tolerance	Glycine betaine-binding protein YehZ	COG1732	M	-	opuC
L_270000016	224	Salt Tolerance	Ktr system potassium uptake protein C	COG0569	P	-	trkA, ktrA
L_270000111	214	Salt Tolerance	Carnitine transport permease protein OpuCB	COG1174	P	-	opuBD
L_270000109	209	Salt Tolerance	Glycine/betaine ABC transporter permease component	COG1174	P	-	opuBD
L_270000403	460	Cold Tolerance	DEAD-box ATP-dependent RNA helicase CshB	COG0513	JKL	MF: Nucleotide binding, Molecular function; BP: Response to stress, Biological process; CC: Cellular component, Intracellular	cshB

L_270001 259	70	Cold Tolerance	Cold shock-like protein	COG1278	K	-	-
L_270001 619	66	Cold Tolerance	Cold shock-like protein CspLA	COG1278	K	-	cspA

Table S10. Putative antibiotic resistance genes annotated in *L. fermentum* 270 against the CARD database

Gene ID	Scaffold	Identify (%)	identify-len	e-value	Coverage (%)	AROid	Gene	Drug Class	Resistance Mechanism
L_270001292	L_270_scaffold22	75.19	391	0	92.43	ARO:3003438	EF-Tu	elfamycin antibiotic	antibiotic target alteration
L_270000988	L_270_scaffold14	68.89	1186	0	100	ARO:3003285	rpoB	rifamycin antibiotic	antibiotic target alteration, antibiotic target replacement
L_270000996	L_270_scaffold14	68.5	692	0	99.86	ARO:3003735	fusA	fusidane antibiotic	antibiotic target alteration
L_270000989	L_270_scaffold14	66.64	1181	0	97.85	ARO:3003291	rpoC	peptide antibiotic	antibiotic target alteration
L_270000057	L_270_scaffold1	65.36	638	0	95.94	ARO:3003315	parE	fluoroquinolone antibiotic	antibiotic target alteration
L_270000627	L_270_scaffold7	62.68	635	0	98.76	ARO:3003301	gyrB	aminocoumarin antibiotic	antibiotic target alteration
L_270000626	L_270_scaffold7	57.78	829	0	93.46	ARO:3003296	gyrA	fluoroquinolone antibiotic	antibiotic target alteration
L_270000058	L_270_scaffold1	55.82	808	0	97.82	ARO:3003311	parC	fluoroquinolone antibiotic	antibiotic target alteration
L_270000308	L_270_scaffold3	55.51	227	3.00E-83	100	ARO:3000838	arlR	disinfecting agents and antiseptics, fluoroquinolone antibiotic	antibiotic efflux
L_270001012	L_270_scaffold14	53.37	178	1.00E-61	100	ARO:3003737	fusE	fusidane antibiotic	antibiotic target alteration
L_270001260	L_270_scaffold21	52.76	923	0	100	ARO:3003729	ileS	mupirocin-like antibiotic	antibiotic target alteration
L_270000041	L_270_scaffold1	52.09	311	2.00E-104	100	ARO:3004153	thyA	salicylic acid antibiotic	antibiotic target alteration
L_270000414	L_270_scaffold4	49.47	190	4.00E-54	97.93	ARO:3003323	pgsA	peptide antibiotic	antibiotic target alteration
L_270000522	L_270_scaffold5	48.74	478	3.00E-150	100	ARO:3003760	cls	peptide antibiotic	antibiotic target alteration
L_270001419	L_270_scaffold26	47.83	230	3.00E-68	93.87	ARO:3002925	vanR	glycopeptide antibiotic	antibiotic target alteration
L_270001753	L_270_scaffold39	47.22	252	3.00E-80	99.21	ARO:3004045	fabI	isoniazid	antibiotic target alteration
L_270000502	L_270_scaffold5	46.64	223	6.00E-68	94.89	ARO:3002928	vanR	glycopeptide antibiotic	antibiotic target alteration
L_270000953	L_270_scaffold13	46.15	481	8.00E-141	97.17	ARO:3003074	cls	peptide antibiotic	antibiotic target alteration
L_270001533	L_270_scaffold30	44.32	264	2.00E-63	60.96	ARO:3004572	LmrS	macrolide antibiotic	antibiotic efflux
L_270001416	L_270_scaffold25	43.53	572	8.00E-166	99.82	ARO:3003899	PtsI	fosfomycin	antibiotic target alteration
L_270000204	L_270_scaffold2	43.46	474	3.00E-127	97.73	ARO:3002813	lmrB	macrolide antibiotic	antibiotic efflux
L_270001208	L_270_scaffold19	43.38	657	5.00E-159	87.13	ARO:3003041	PBP1a	monobactam	antibiotic target alteration
L_270000220	L_270_scaffold2	43.15	387	7.00E-113	93.7	ARO:3001329	mdtG	macrolide antibiotic	antibiotic efflux
L_270000040	L_270_scaffold1	42.99	642	4.00E-178	100	ARO:3003986	TaeA	macrolide antibiotic	antibiotic efflux
L_270001960	L_270_scaffold53	42.6	831	0	96.4	ARO:3003770	mprF	peptide antibiotic	antibiotic target alteration
L_270000034	L_270_scaffold1	42.33	352	6.00E-90	85.85	ARO:3004721	rpsA	pyrazinamide	antibiotic target alteration
L_270001607	L_270_scaffold33	42.01	269	6.00E-54	100	ARO:3002986	bacA	peptide antibiotic	antibiotic target alteration

L_270001760	L_270_scaffold39	41.98	243	2.00E-59	100	ARO:3004049	-	-	-
L_270000919	L_270_scaffold12	41.79	268	1.00E-59	100	ARO:3004097	CdsA	peptide antibiotic	antibiotic target alteration
L_270000503	L_270_scaffold5	41.61	620	7.00E-166	100	ARO:3003794	walK	peptide antibiotic	antibiotic target alteration
L_270000580	L_270_scaffold6	41.00	422	4.00E-96	100	ARO:3003776	murA	fosfomycin	antibiotic target alteration
L_270000950	L_270_scaffold13	39.75	473	2.00E-111	81.13	ARO:3003948	efrA	macrolide antibiotic	antibiotic efflux
L_270001759	L_270_scaffold39	38.72	390	2.00E-82	96.53	ARO:3003463	kasA	isoniazid	antibiotic target alteration
L_270000497	L_270_scaffold5	38.13	619	5.00E-151	91.43	ARO:3003797	YybT	peptide antibiotic	antibiotic target alteration
L_270001630	L_270_scaffold33	38.12	480	9.00E-105	98.56	ARO:3004572	LmrS	macrolide antibiotic	antibiotic efflux
L_270001651	L_270_scaffold34	37.54	570	4.00E-129	98.95	ARO:3003899	PtsI	fosfomycin	antibiotic target alteration
L_270000190	L_270_scaffold2	36.67	300	5.00E-55	91.74	ARO:3002948	vanH	glycopeptide antibiotic	antibiotic target alteration
L_270000951	L_270_scaffold13	35.94	601	9.00E-129	98.04	ARO:3000025	patB	macrolide antibiotic	antibiotic efflux
L_270000599	L_270_scaffold6	35.88	379	1.00E-81	97.93	ARO:3003551	emeA	fluoroquinolone antibiotic	antibiotic efflux
L_270001602	L_270_scaffold32	35.76	646	2.00E-137	99.84	ARO:3003746	optrA	macrolide antibiotic	antibiotic target protection
L_270000517	L_270_scaffold5	35.71	280	1.00E-52	84.84	ARO:3002942	vanH	glycopeptide antibiotic	antibiotic target alteration
L_270000294	L_270_scaffold3	35.58	683	4.00E-119	98.41	-	-	-	-
L_270001420	L_270_scaffold26	35.56	270	1.00E-52	71.05	ARO:3002936	vanS	glycopeptide antibiotic	antibiotic target alteration
L_270000981	L_270_scaffold13	35.31	354	1.00E-67	93.89	ARO:3003970	D-Ala-D-Ala ligase	glycopeptide antibiotic	antibiotic target alteration
L_270000569	L_270_scaffold6	35.14	370	7.00E-59	99.19	ARO:3002972	vanT	glycopeptide antibiotic	antibiotic target alteration
L_270000219	L_270_scaffold2	34.81	451	6.00E-77	95.75	ARO:3002813	lmrB	macrolide antibiotic	antibiotic efflux
L_270001306	L_270_scaffold22	34.37	323	4.00E-58	97.28	ARO:3002945	vanH	glycopeptide antibiotic	antibiotic target alteration
L_270002055	L_270_scaffold66	33.65	419	2.00E-77	95.01	ARO:3004573	AbaF	macrolide antibiotic	antibiotic efflux
L_270001380	L_270_scaffold24	33.46	508	8.00E-89	100	ARO:3003746	optrA	macrolide antibiotic	antibiotic target protection
L_270000307	L_270_scaffold3	32.81	384	8.00E-64	76.64	ARO:3000839	arlS	macrolide antibiotic	antibiotic efflux
L_270000938	L_270_scaffold12	32.76	409	6.00E-72	90.88	ARO:3002813	lmrB	macrolide antibiotic	antibiotic efflux
L_270000344	L_270_scaffold3	32.57	614	2.00E-70	86.96	ARO:3003041	PBP1a	monobactam	antibiotic target alteration
L_270001271	L_270_scaffold21	32.52	741	2.00E-115	100	ARO:3003043	PBP2x	monobactam	antibiotic target alteration
L_270001159	L_270_scaffold18	30.15	461	6.00E-70	100	ARO:3003889	GlpT	fosfomycin	antibiotic target alteration
L_270001212	L_270_scaffold19	29.60	642	4.00E-88	99.68	ARO:3000193	tetT	tetracycline antibiotic	antibiotic target protection
L_270000823	L_270_scaffold10	29.26	417	2.00E-56	85.10	ARO:3003805	gshF	peptide antibiotic	antibiotic target alteration
L_270000779	L_270_scaffold9	29.12	697	4.00E-68	100	ARO:3000535	macB	macrolide antibiotic	antibiotic efflux
L_270000373	L_270_scaffold3	28.95	494	4.00E-59	100	ARO:3002813	lmrB	macrolide antibiotic	antibiotic efflux
L_270000826	L_270_scaffold10	27.39	533	7.00E-62	98.52	ARO:3003986	TaeA	macrolide antibiotic	antibiotic efflux

Table S11. Mobile Genetic Elements Identification Results

MGE identifier	Element name	Element type	Prediction method	Length (bp)	Percent identity (%)	Coverage (%)	E-value	Scaffold	Start position (bp)	End position (bp)
15	IS1165	Insertion sequence	alignment to reference	1462	99.04	94.14	0	L_270_scaffold51	274	1735
4	IS153	Insertion sequence	alignment to reference	596	98.83	52.88	0	L_270_scaffold19	315	910
5	IS153	Insertion sequence	alignment to reference	547	97.27	48.54	0	L_270_scaffold19	1119	1665
6_4_5	cn_1351_IS153	Composite transposon	inferred	1351	98.83	52.88	0	L_270_scaffold19	314	1665
10	IS153	Insertion sequence	alignment to reference	597	96.15	52.80	0	L_270_scaffold35	2266	2862
11	IS153	Insertion sequence	alignment to reference	433	93.36	38.42	0	L_270_scaffold35	3085	3517
12_10_11	cn_1252_IS153	Composite transposon	inferred	1252	96.15	52.80	0	L_270_scaffold35	2265	3517
